# Habitat-Based Radiomics for Predicting Visceral Pleural Invasion in Subpleural Nodules with Solid Component on Low-Dose CT: A Multicenter Study

**DOI:** 10.3390/diagnostics16081191

**Published:** 2026-04-16

**Authors:** Yu Long, Xiaoyu Li, Yong Li, Yongji Zheng, Wei Lin, Peng Zhou, Jieke Liu

**Affiliations:** 1 Department of Radiology, Sichuan Clinical Research Center for Cancer, Sichuan Cancer Hospital & Institute, Sichuan Cancer Center, University of Electronic Science and Technology of China, Chengdu 610041, China; longyu1301@foxmail.com (Y.L.); lixxyu@foxmail.com (X.L.); liyong_scszlyy@foxmail.com (Y.L.); penghyzhou@126.com (P.Z.); 2 Department of Radiology, Deyang People’s Hospital, Deyang 618000, China; zheng.yongji@foxmail.com; 3 Department of Radiology, Chengdu First People’s Hospital, Chengdu 610041, China; linwei_cfph@foxmail.com

**Keywords:** habitat analysis, visceral pleural invasion, low-dose computed tomography, adenocarcinoma of lung

## Abstract

**Objectives**: Our objectives were to develop and validate the habitat model based on low-dose computed tomography (LDCT) for noninvasive prediction of the visceral pleural invasion (VPI) in subpleural nodules with solid component. **Methods**: A total of 313 patients with subpleural lung adenocarcinoma nodules from three centers were retrospectively enrolled and divided into training (*n* = 192), validation (*n* = 82), and external test (*n* = 39) sets. All patients underwent preoperative LDCT scan. The habitat model was constructed using unsupervised clustering to partition each tumor into three distinct habitats, from which radiomic features were extracted and selected. Its diagnostic performance was compared with a whole-lesion radiomic model and radiological model. Statistical analysis included receiver operating characteristic (ROC) analysis and DeLong test. **Results**: The habitat model significantly outperformed both the radiomic and radiological models across the validation and external test sets, with areas under the ROC curve of 0.893 and 0.908, respectively (all *p* < 0.05). In contrast, the radiomic model achieved 0.833 and 0.772, while the radiological model yielded 0.746 and 0.624. The corresponding software tool has been made publicly available to facilitate broader clinical application. **Conclusions**: The habitat imaging model based on LDCT effectively predicts the VPI in subpleural lung adenocarcinoma by quantifying intratumoral spatial heterogeneity and demonstrates promising diagnostic performance compared to conventional radiomic and radiological methods. This approach offers a noninvasive preoperative tool to assist in risk stratification and guide personalized therapeutic decision-making for subpleural nodules detected during lung cancer screening.

## 1. Introduction

Recently, the detection rate of nodules has been on the rise, primarily attributed to the increased application of low-dose computed tomography (LDCT) [1]. Compared with standard-dose CT (SDCT), LDCT offers notable clinical advantages in lung cancer screening, including substantially reduced radiation exposure, improved patient compliance, and proven efficacy in reducing lung cancer mortality in high-risk populations [2,3]. In the context of lung cancer screening, attention should be paid to subpleural nodules, due to their higher association with visceral pleural invasion (VPI) [4]. VPI is defined as a criterion for T2 staging in the Tumor–Node–Metastasis (TNM) system, regardless of tumor size [5], and serves as an indicator for the potential lymphatic vessel invasion and lymph node metastasis [6], exhibiting reduced overall survival rates [7] and an elevated risk for recurrence [8].Thus, accurate evaluation of VPI could assist clinicians stratifying the risk of subpleural nodules and guiding treatment strategies.

Given the limitations of preoperative biopsy and intraoperative frozen section in accurately identifying VPI due to sampling constraints, previous studies used preoperative computed tomography (CT) findings to assess the VPI status, such as direct contact with the pleura (pleural attachment) and indirect contact through a linear strand (pleural tags). Several CT features have been proposed as potential indicators, including a convex tumor-pleural border with a perpendicular or blunt angle [9], pleural tags with retraction [10], one soft tissue cord-like pleural tag [11], and the arch-shaped linear tag between the tumor and the pleura (bridging tag sign) [12]. However, the reported CT imaging features exhibit significant heterogeneity and are subject to inherent interpretation variability, which limits their clinical utility. Consequently, previous radiomics has garnered considerable interest [13,14,15]. Nevertheless, these whole-lesion radiomic studies assumed feature homogeneity within the tumor, overlooking the phenotypic heterogeneity which present across different tumor subregions by anatomical location, vascular interactions, and host immune responses [16]. This spatial heterogeneity directly underpins the emerging rationale for habitat imaging.

Habitat analysis characterizes the heterogeneity within tumor region by employing unsupervised clustering to group voxels with similar attributes. This methodology offers a powerful framework for identifying distinct tumor subregions, termed “habitats”, that may correspond to unique biological behaviors. Radiomic features extracted from such habitats have demonstrated diagnostic value across multiple applications in lung adenocarcinoma, including predicting the high-grade components [17], lymphovascular invasion [18], and lung adenocarcinoma invasiveness [19]. Previous literature revealed that the image acquisition and reconstruction parameters could affect the reproducibility of radiomic features [20]. Consequently, the radiomic models based on SDCT may not be applicable to LDCT in lung cancer screening. In addition, our previous study demonstrated that LDCT-based habitat models achieved high efficacy in predicting the malignant degree of lung adenocarcinomas [21]. Thus, we hypothesized that habitat imaging holds potential to support the preoperative evaluation of VPI in lung adenocarcinoma.

Therefore, this study aimed to develop and validate a habitat imaging model using LDCT to predict VPI in subpleural nodules with solid component. Of note, pure ground-glass nodule was excluded owing to it hardly existing VPI [22,23], and showed excellent prognosis after sublobar resection.

## 2. Materials and Methods

### 2.1. Patients

This multicenter, retrospective study, conducted in accordance with the Standards for Reporting Diagnostic Accuracy (STARD) statement [24], was granted a waiver of ethical approval by the Ethics Committees of all participating hospitals.

The patient inclusion criteria included the following: (1) adenocarcinoma underwent surgery and confirmed by pathology; (2) subpleural nodules, which were in contact with the surface of costal, mediastinal, or diaphragmatic pleura surface or presence of pleural tags; (3) part-solid or solid nodules; (4) thin-slice chest LDCT scan (slice thickness ≤ 1 mm) performed within 2 weeks before surgery; (5) a complete pathological report detailing VPI status. The exclusion criteria encompassed the following: (1) diameter ≥ 3 cm; (2) poor LDCT image quality. In cases of multiple resected nodules per patient, the nodule exhibiting the largest maximum diameter was retained for analysis.

**Figure 1:**
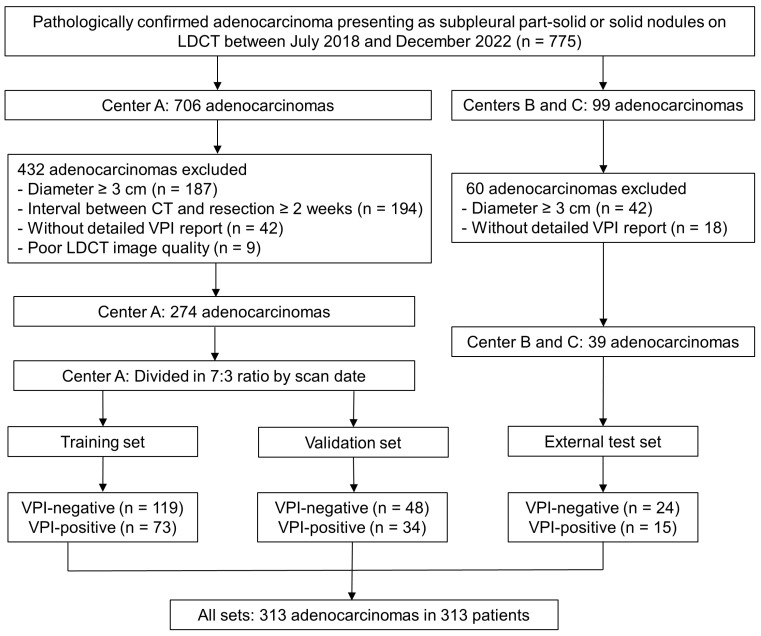
The flowchart of patient inclusion and exclusion. LDCT, low-dose CT; VPI, visceral pleural invasion.

In total, this study comprised 313 patients (median age, 59 years [interquartile ranges, 52–68 years]; 188 females) from three participating centers. Patients from a specialized cancer hospital (Center 1: July 2018–December 2022) were divided into the training (*n* = 192) and validation (*n* = 82) sets in a 7:3 ratio based on scan date. An external test set (*n* = 39) comprised patients from two tertiary hospitals (Center 2 and Center 3: February 2021–December 2022). Patient selection is detailed in the flowchart (Figure 1).

### 2.2. Histopathological Evaluation

The histopathologic diagnosis of lung adenocarcinoma was performed in accordance with the 2021 WHO classification (5th edition) of lung tumors [5]. Each surgical specimen underwent Elastica–Masson and elastic staining to determine the VPI status. Positive VPI indicates tumor penetration beyond the elastic layer of the visceral pleura, categorized as PL1 (where the tumor extends beyond this elastic boundary but remains unexposed on the pleural surface) and PL2 (where the tumor is visible on the pleural surface without invading surrounding structures) [25].

### 2.3. Image Acquisition and Harmonization of CT Images Across Centers

Patients from Center 1 underwent non-contrast chest LDCT scans using a 64-detector SOMATOM Definition Flash CT scanner, with a mean radiation dose of 0.55 mSv (±0.12). Scans in center 2 and 3 were acquired from three different multi-detector CT, with a mean radiation dose of 1.02 mSv (±0.36). Supplementary Table S1 summarizes the acquisition and reconstruction parameters.

To minimize scanner-related variance, noise, and reduced image quality in LDCT, all images were harmonized according to the Imaging Biomarker Standardization Initiative [26]. After resampling to 1 × 1 × 1 mm^3^ voxels (linear interpolation), and discretizing into 25 bins to suppress noise and accelerate radiomic feature extraction, both radiomic and habitat features were harmonized across scanners using the ComBat method to minimize inter-scanner variability, which was available at https://github.com/Jfortin1/ComBatHarmonization (accessed on 26 July 2025) [27,28].

The implementation of ComBat harmonization was detailed in the Supplementary Materials, including (1) preprocessing steps prior to harmonization; (2) full list of radiomic features harmonized; (3) definition of five batches based on center–scanner combinations; (4) mathematical formulation of the ComBat model; (5) input and parameters of for ComBat; (6) Key implementation details; and (7) validation of harmonization effectiveness.

In addition, to quantitatively assess the effectiveness of harmonization, we computed the proportion of variance in each radiomic feature explained by batch (R^2^) before and after ComBat, averaged across all features in the training set. The mean R^2^ decreased from 1.17% before harmonization to 0.49% after harmonization, representing a 58.2% relative reduction. A paired *t*-test showed the reduction was statistically significant (*p* < 0.001). These results confirm that ComBat pipeline effectively reduced inter scanner variability in the training set.

The detailed R^2^ results and a boxplot of the per feature R^2^ distributions have been added to the Supplementary Materials and Supplementary Figure S5.

### 2.4. Radiological Model Development

**Figure 2:**
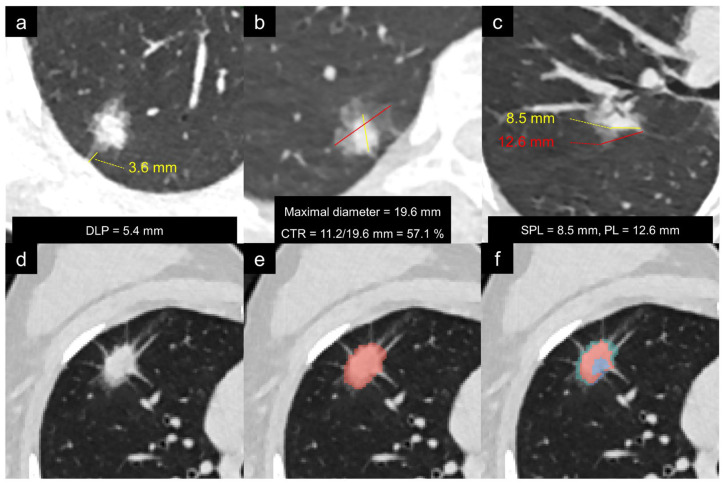
Definitions of radiological features and representative examples of radiomic and habitat-based segmentations. (**a**) Minimum distance between lesion and pleura (DLP). (**b**) Consolidation-to-tumor ratio (CTR) based on the maximal diameter of nodule (red line) and solid component (yellow line). (**c**) The solid component–pleural contact length (SPL, yellow line) and lesion–pleural contact length (PL, red line). (**d**) Original low-dose CT image. (**e**) Representative result of the whole lesion radiomic segmentation. (**f**) Representative result of three habitat segmentations: habitat 1 (green) at the peripheral edge adjacent to the pleura, habitat 2 (pink) in the transitional zone, and habitat 3 (blue) in the central core.

Two radiologists (Yong Li, 4 years of experience; J.L., 9 years of experience), blinded to clinical and pathological data, independently assessed the radiological features. Quantitative metrics included maximal diameter, solid component diameter, the shortest distance from the lesion to the pleura (DLP), tumor–pleural contact length (PL), and solid tumor-pleural contact length (SPL). Qualitative features evaluated were lobulation, spiculation, air bronchogram sign, vascular convergence sign, and tumor–pleural relationship (pleural tags and attachment). The consolidation-to-tumor ratio (CTR) was calculated as (solid component diameter/maximal diameter) × 100% [29]. The definitions of radiological features are detailed in Figure 2a–c. Inter-observer agreement for quantitative features was evaluated using the intra-class correlation coefficient (ICC), with values > 0.80 considered to indicate satisfactory consistency. The mean measurements from the two radiologists were used for all subsequent analyses. For the remaining semantic features, any discrepancies were resolved through consensus.

Univariate and multivariate logistic regression analyses, with the stepwise backward elimination, were used to identify the optimal radiological characteristics, which were subsequently used to construct the radiological model, and its output probability for VPI-positive classification was defined as the radiological signature.

### 2.5. Radiomics Feature Selection and Model Development

A deep-learning software automatically detected and segmented each nodule to generate a three-dimensional VOI. This software (uAI Research Platform, United Imaging Intelligence, version R001, Shanghai, China) integrates deep learning-based algorithms for automatic pulmonary nodule detection and three-dimensional segmentation [30,31]. The platform provides a comprehensive toolset for medical image analysis, including automated lesion segmentation, quantification, and radiomic feature extraction, and has been validated in prior studies on lung nodule characterization [21,32]. The resulting segmentations, illustrated in Figure 2d,e, were subsequently reviewed by two radiologists (Yong Li and J. L.), and any suboptimal VOI boundaries were manually refined through consensus. A total of 106 radiomic features were extracted from the VOIs using PyRadiomics (Supplementary Table S2) and normalized via Z-score transformation. Feature selection within the training set was performed in four sequential steps to identify the optimal features for model construction: (1) exclusion of highly correlated features (Spearman’s ρ > 0.9); (2) selection of features demonstrating significant inter-group differences via the Mann–Whitney U test; (3) refinement using the minimum redundancy maximum relevance (mRMR) algorithm to retain the top 10 features [3]; and (4) final optimization with the least absolute shrinkage and selection operator (LASSO) regression [33].

Multivariate logistic regression, with the stepwise backward elimination, was employed to develop the radiomic model, and its output probability for VPI-positive classification was defined as the radiomic signature. This signature was then validated on both the validation and external test sets.

### 2.6. Habitat Generation and Model Development

Habitat generation was performed through a three-stage clustering process applied to the VOIs in the training set. First, image intensities were normalized to a mean of 0 and variance of 1, with pixel values constrained to the lung window range of −1250 to 250 HU (width: 1500 HU; level: −500 HU). Subsequently, each VOI was segmented into 50 superpixel blocks using the Simple Linear Iterative Clustering (SLIC) algorithm [34,35]. Finally, ten first-order statistical features were extracted from each superpixel based on gray-level histogram distributions [36], and all superpixels were clustered using the K-means algorithm to define distinct habitat regions. In addition, the K-means clustering data were saved to ensure consistent application of the clustering method to the validation and external test set, thereby enhancing the reproducibility and generalizability of the study. The optimal number of clusters was determined using the Davies–Bouldin (DB) index, with cluster separability visually confirmed via Principal Component Analysis (PCA) [37].

The subsequent feature extraction and selection were performed individually for each habitat, following the identical pipeline used for radiomic model, as outlined in the previous section. The final habitat model integrates the output probabilities from each habitat through the logistic regression, and the final resulting output probability was defined as the habitat signature, and was also validated on both the validation and external test sets. However, not all samples were guaranteed to contain the same number of clusters. To address this, missing habitat regions were imputed using the missForest algorithm [38].

### 2.7. Statistical Analysis

Continuous variables are presented as median (interquartile range, IQR), and categorical variables as frequencies (percentages). Group comparisons were performed using the Mann–Whitney U test for continuous variables and Fisher’s exact test for categorical variables, with a two-tailed *p* value < 0.05 considered statistically significant. All statistical analyses were performed in Python (version 3.10.1).

The multicollinearity of selected features for the radiological, whole-lesion radiomic, and habitat models was evaluated using the variance inflation factor (VIF). The performance of the three models was assessed utilizing receiver operating characteristic (ROC) curve analysis, with results reported as the area under the curve (AUC) and its 95% confidence interval (CI), along with sensitivity, specificity, positive predictive value (PPV), and negative predictive value (NPV). AUC comparisons were performed using the DeLong test [2]. The DeLong test is a nonparametric method that accounts for the correlated nature of ROC curves derived from the same set of patients, comparing the equality of two AUCs by estimating the covariance matrix between the curves. All DeLong tests were two-sided, and a *p* value < 0.05 was considered statistically significant. Analyses were conducted using the delong_roc_test function implemented in Python. The clinical utility was assessed via decision curve analysis (DCA), and calibration of three models were evaluated using the brier score and visualized using the calibration curves.

A complete analytical pipeline, including the habitat model and clustering algorithm developed in our training set, was implemented as a downloadable software application. The source code, pretrained model files, and detailed documentation have been made publicly available to ensure reproducibility and facilitate external validation.

## 3. Results

### 3.1. Patient Characteristics

Table 1 presents the baseline characteristics of the study population, which was stratified into three distinct datasets: training set (119 VPI-negative and 73 VPI-positive), validation set (48 VPI-negative and 34 VPI-positive), and external test set (24 VPI-negative and 15 VPI-positive).

As shown in Table 1, significant differences were found between the VPI-negative and VPI-positive groups for age (*p* = 0.012), maximal diameter (*p* = 0.039), and several other features including solid component diameter, nodule type, CTR, DLP, PL, SPL, and the tumor-pleura relationship (all *p* < 0.001). Notably, no significant differences were found for lobulation, spiculation, air bronchogram, or vascular convergence sign (all *p* > 0.05). As for dataset comparability, analyses revealed no significant differences in nodule characteristics between the training set and the validation or external test sets (all *p* > 0.05)**.**

### 3.2. Radiological Model

The ICCs were 0.863, 0.812, 0.886, 0.834, and 0.820 for maximal diameter, solid component diameter, DLP, PL, and SPL, respectively, indicating satisfactory agreements between two radiologists. In the univariable logistic analysis, maximal diameter, solid component diameter, nodule type, CTR, and SPL were significantly associated with VPI (Supplementary Table S3). After multivariate logistic analysis, the maximal diameter, solid component diameter and SPL were remained as independent predictors of VPI in the training set. However, maximal diameter was excluded due to its moderate correlation with solid component diameter (Spearman’s ρ = 0.572, *p* < 0.001) and its inferior diagnostic performance. No significant multicollinearity issues exist among the selected features (Supplementary Table S5).

Thus, the two remaining radiological features were used to develop the radiological model (Table 2). The radiological signature was derived using the following equation: ln[*p*/(1 − *p*)] = −3.164 + 0.162 × solid component diameter + 0.063 × SPL. Here, *p* denoted the predicted probability, and a *p* value > 0.348 was defined as indicative of VPI-positive status.

### 3.3. Radiomic Model

Six radiomic features were retained following the LASSO selection method. The results and corresponding coefficient value of each selected feature are detailed in Supplementary Figure S1. After univariable and multivariate logistic regression, three radiomic features were retained as the independent predictors of VPI to develop the radiomic model in the training set (Supplementary Table S3) (Table 2). No significant multicollinearity issues exist among the selected features (Supplementary Table S5). The radiomic signature was derived using the following equation: ln [*p*/(1 − *p*)] = −0.876 − 1.036 × GLCM_ClusterShade + 0.621 × GLDM_LargeDependenceEmphasis − 1.864 × GLDM_LargeDependenceLowGrayLevelEmphasis. Here, *p* denoted the predicted probability, and a *p* value > 0.358 was defined as indicative of VPI-positive status. To calculate the standardized (Z-score) values for the three features used, their original-scale mean and standard deviation are provided in Supplementary Table S4.

### 3.4. Habitat Model

**Figure 3:**
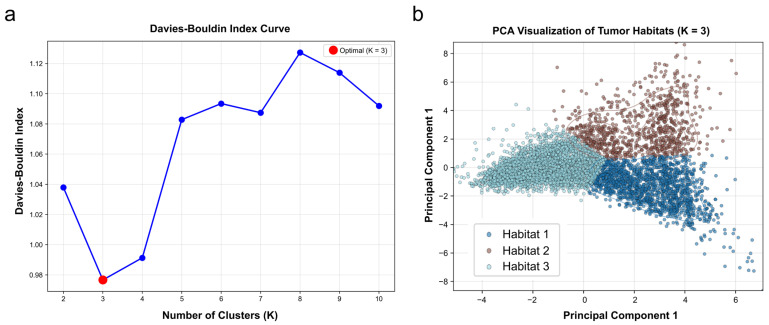
Determination of the optimal cluster number and validation of cluster separability. (**a**) The Davies–Bouldin index quantifies the ratio of within-cluster to between-cluster distances, where a smaller value indicates better clustering. Hence, based on the curve minimum, three was selected as the optimal number of habitats for the present analysis. (**b**) Cluster separability was visually confirmed via principal component analysis.

The Davies–Bouldin (DB) index was calculated to identify the optimal number of clusters, with values corresponding to K ranging from 2 to 10 being 1.038, 0.991, 0.977, 1.083, 1.093, 1.087, 1.127, 1.114, and 1.092 (Figure 3a). Since the minimal DB value occurred at K = 3, three was selected as the optimal number of habitats. Cluster separability was visually confirmed through Principal Component Analysis (PCA), which showed clear discrimination among the three habitats (Figure 3b). Representative examples of the resulting three-habitat segmentations are illustrated in Figure 2f. Of note, a total of 24 missing habitat regions were imputed using the missForest algorithm. In the training set, 13 out of 192 samples (6.8%) required imputation for at least one missing habitat; in the validation set, 7 out of 82 (8.5%); and in the external test set, 4 out of 39 (10.3%). Cluster 2 was the most frequently missing habitat across all datasets (four in the training set; three in the validation set; and two in the external test set).

From each habitat, only the “shape_voxel_volume” feature was retained, while all other shape features were excluded. This resulted in a final set of 279 features, derived from 93 features per habitat. Following the LASSO feature selection, nine radiomic features were retained, and the corresponding results and coefficient value of each selected feature are detailed in Supplementary Figure S1. After univariable and multivariate logistic regression, five habitat features were retained as the independent predictors of VPI to develop the habitat model (Supplementary Table S3) (Table 2). No significant multicollinearity issues exist among the selected features (Supplementary Table S5). The habitat signature was calculated by the following equation: ln[*p*/(1 − *p*)] = −1.032 − 0.706 × Habitat1_Firstorder_Skewness + 0.504 × Habitat1_GLDM_SmallDependenceEmphasis − 1.234 × Habitat2_NGTDM_Busyness − 0.826 × Habitat3_GLCM_ClusterProminence − 0.712 × Habitat3_NGTDM_Contrast. Here, *p* denoted the predicted probability, and a *p* value > 0.476 was defined as indicative of VPI-positive status. Since all habitat features were Z-score normalized, their original-scale mean and standard deviation are provided in Supplementary Table S4.

### 3.5. Diagnostic Performance

**Figure 4:**
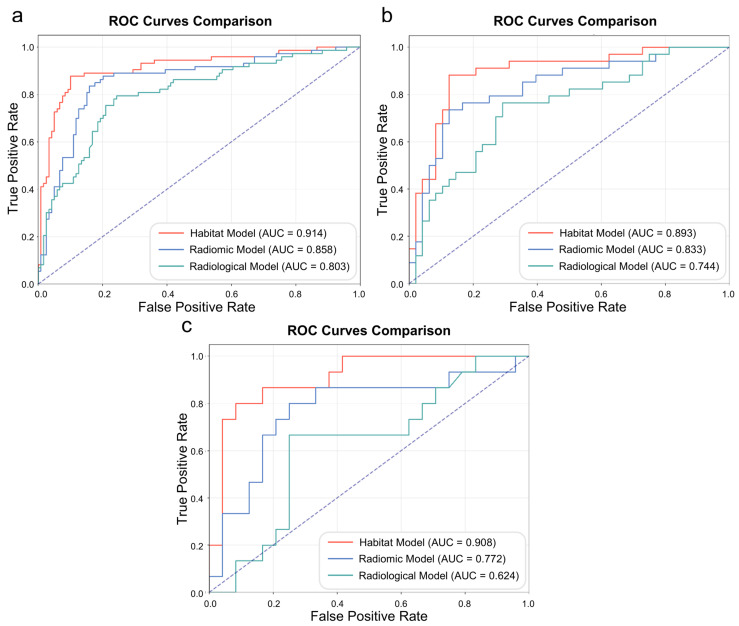
Receiver operating characteristic curves of the radiological, radiomic, and habitat models. (**a**) Training set. (**b**) Validation set. (**c**) External test set.

Figure 4 presents the ROC curves of the habitat, whole-lesion radiomic, and radiological models, which achieved the AUCs in the training set of 0.914, 0.858, and 0.803; in the validation set of 0.893, 0.833, and 0.746; and in the external test set of 0.908, 0.772, and 0.624, respectively. Table 3 summarizes the models’ performance in three datasets, including the AUC, sensitivity, specificity, PPV, and NPV. The corresponding confusion matrices are detailed in Supplementary Figure S2.

As detailed in Table 4, pairwise comparisons revealed that the habitat model was statistically superior to the radiomic model, with significantly higher AUCs in the validation (*p* = 0.042) and external test sets (*p* = 0.015). Furthermore, the habitat model also significantly outperformed the radiological model in both the validation and external test sets (both *p* < 0.001). The DCA and calibration curves of three models in three datasets are provided in the Supplementary Figure S3 and Supplementary Figure S4, respectively. In the training set, the Brier scores were 0.102 for the habitat model, 0.145 for the radiomic model, and 0.171 for the radiological model. In the validation set, they were 0.119, 0.159, and 0.178, respectively. In the external test set, they were 0.077, 0.156, and 0.139, respectively. The Brier scores showed acceptable calibration performance across three datasets.

The STARD checklist is provided in the Supplementary Table S6. The radiomics quality score of this study was 24, suggesting a favorable quality (Supplementary Table S7).

### 3.6. Clinical Application

To facilitate broader clinical application, we developed an integrated, user-friendly software package for predicting VPI in patients with subpleural nodules with solid component through a fully automated computational pipeline. The software implements a sequential workflow that includes image preprocessing (e.g., resampling and normalization), 3D SLIC superpixel segmentation, habitat clustering based on the K-means clustering data developed from our training set, radiomic feature extraction per habitat, and logistic regression-based prediction, all within an intuitive graphical user interface. Both the software and the pretrained k-means clustering model are publicly available at https://github.com/mzi969/Image-Analysis-Pipeline-for-VPI-on-LDCT (accessed on 15 November 2025), and a detailed software manual was provided in the Supplementary Materials.

## 4. Discussion

This multicenter study developed and validated a habitat imaging model based on LDCT to predict VPI in subpleural nodules with solid component. Our results demonstrated that the habitat model significantly outperformed both the whole-lesion radiomic model and conventional radiological models in diagnostic performance across the validation and external test sets. This superior performance indicates that habitat analysis more effectively captures the spatial tumor heterogeneity underlying VPI, facilitated by the software tool we developed, highlighting its potential clinical utility for risk stratification and treatment decision-making in patients with subpleural nodules detected in lung cancer screening.

Subpleural nodules are fundamentally distinct from intraparenchymal nodules due to their anatomical adjacency to the pleura [9]. Accurate identification of VPI status in these nodules is critical for clinical management, as VPI is associated with increased postoperative recurrence and lymph node metastasis following sublobar resection [6,39]. Currently, definitive diagnosis relies on postoperative pathological assessment, which inevitably delays surgical decision-making. To address this, CT-based predictive models have been investigated. Our results support the established view that imaging biomarkers such as solid component diameter and SPL are indicative of elevated VPI risk. Nevertheless, conventional approaches relying on semantic radiological signs (e.g., pleural tags, pleural attachment) and basic quantitative measurements are constrained by inter-observer variability and limited diagnostic accuracy, as reflected in the declining performance of our radiological model across validation and external test sets, with the AUC values of 0.803 in the training set, 0.746 in the validation set, and 0.624 in the external test set.

Prior studies have employed radiomics models to predict VPI [13,40]. However, features derived from matrices such as Gray-Level Co-occurrence and Gray-Level Dependence, while informative, were consistently outperformed by our habitat model. This suggests that treating the tumor as a radiologically homogeneous entity may obscure critical biological information residing in distinct intratumoral subregions, thereby limiting predictive capacity [19,41,42]. The core strength of the habitat model lies in its ability to identify and quantify intratumoral heterogeneity in an unsupervised manner. Through clustering analysis, we partitioned each nodule into three habitats with distinct imaging phenotypes. Features were subsequently extracted and selected from each habitat individually, and the final integrated habitat model combined unique information from multiple subregions. The habitat model’s robust and superior performance across three datasets indicates that different habitats may reflect region-specific biological processes during tumor growth and invasion [36]. For instance, subregions adjacent to the pleural interface might harbor information more directly related to invasive behavior. Our approach moves beyond traditional “whole-lesion” radiomics analysis, offering a more granular perspective for understanding tumor heterogeneity and its association with clinical outcomes like VPI.

Notably, the superior performance of the habitat model stems from its capacity to capture intratumoral spatial heterogeneity. Habitat 1, characterized by negative skewness and fine-textured homogeneity, represents the metabolically active, proliferating tumor periphery [43]. Its proximity to the pleural surface facilitates direct invasion in subpleural nodules [10]. Habitat 2, marked by elevated NGTDM_Busyness, captures complex tissue interfaces corresponding to the tumor pleura transition zone with desmoplastic stroma, inflammatory infiltration, irregular glandular infiltration, and disrupted elastic lamina [5,44]. Notably, we found the cluster 2 was the most frequently missing habitat across all datasets. In subpleural nodules that do not directly about the pleura or have only subtle pleural tags, such an invasive interface may be absent or too thin to be resolved on LDCT, resulting in the non-detection of cluster 2. Habitat 3, with low GLCM_ClusterProminence and reduced NGTDM_Contrast, reflects homogeneous low-attenuation areas consistent with necrosis or mucin pools. All these indicate that VPI arises from combined effects of spatially heterogeneous subregions, a complexity effectively quantified through habitat analysis [45].

Beyond methodological advantages, this study carries significant clinical implications. Our model was specifically developed for LDCT, the standard imaging modality in lung cancer screening, enabling its direct integration into existing screening workflows. Through the software tool we developed and made publicly available, clinicians can conveniently assess VPI risk in subpleural nodules, thereby facilitating more personalized preoperative treatment planning. For patients suspected of being VPI-positive, more aggressive interventions such as lobectomy and adjuvant chemotherapy may be warranted, whereas patients suspected of being VPI-negative could avoid overtreatment, particularly those eligible for sublobar resection. In addition, LDCT inherently involves higher image noise and reduced spatial resolution compared with SDCT, which may affect the stability and reproducibility of radiomic features and habitat clustering. In particular, texture features that depend on local intensity variations (e.g., GLCM, NGTDM) are more susceptible to noise, whereas first-order statistical features (mean, skewness, etc.) are relatively robust [26]. For habitat analysis, elevated noise could influence superpixel segmentation and the subsequent K-means clustering, potentially leading to variability in habitat assignment. Inter-scanner variability can also shift the absolute intensity values assigned to identical tissues, potentially causing pixels to cross the predefined habitat thresholds and be re-classified into a different habitat [46]. To address this, we implemented an image processing pipeline that harmonized intensity distributions across scanners, thereby ensuring high reliability and generalizability of the derived habitat features in multicenter data.

This study has several limitations. First, its retrospective design may introduce selection bias, which necessitates prospective validation to confirm our findings. Second, although developed using a multicenter dataset, the external test set (*n* = 39) was relatively small, resulting in wide confidence intervals; future studies with larger external cohorts from diverse clinical settings are needed to further substantiate the model’s clinical utility. Third, the habitat model was derived from LDCT reconstructed with smooth kernels, which may limit its applicability to datasets acquired or processed under different protocols. Future studies should systematically evaluate the impact of varying noise levels and contrast on habitat reproducibility using phantom or digital phantom data. Fourth, the imputation of missing habitats is an inherent limitation of habitat-based approaches, because the unsupervised clustering algorithm may not always identify a fixed number of distinct subregions in every tumor. Future studies with larger sample sizes and higher-resolution CT protocols may reduce the occurrence of missing habitats.

## 5. Conclusions

In this study, by quantifying intratumoral spatial heterogeneity, the habitat model demonstrated significantly superior diagnostic performance compared to conventional whole-lesion radiomic and visually assessed radiological models. Habitat imaging offers a novel and effective framework for noninvasive assessment of tumor heterogeneity and the prediction of VPI in subpleural nodules with solid component. The accompanying publicly available software tool further enhances the accessibility, reproducibility, and translational potential of this study.

## Figures and Tables

**Table 1:** Patient characteristics in the training set, validation set, and external test set.

Characteristic	Training Set (*n* = 192)		*p* ^a^	Validation Set (*n* = 82)	*p* ^b^	External Test Set (*n* = 39)	*p* ^b^
	VPI-Negative (*n* = 119)	VPI-Positive (*n* = 73)					
Age	59 (52, 67)	66 (57, 70)	0.012 *	61 (54, 68)	0.646	62 (57, 66)	0.743
Gender			0.070		0.592		0.721
Male	41 (34.5%)	35 (47.9%)		36 (43.9%)		17 (43.6%)	
Female	78 (65.5%)	38 (52.1%)		46 (56.1%)		22 (56.4%)	
Maximal diameter (mm)	19.6 (15.1, 23.4)	21.0 (17.3, 24.6)	0.039 *	18.2 (14.9, 22.9)	0.124	18.3 (14.8, 23.4)	0.386
Solid component diameter (mm)	10.5 (7.1, 14.2)	18.9 (14.7, 23.0)	<0.001 *	12.3 (7.9, 20.3)	0.507	13.4 (7.4, 16.3)	0.282
Nodule type			<0.001 *		0.182		0.194
Part solid nodule	92 (77.3%)	24 (32.9%)		42 (51.2%)		24 (61.5%)	
Solid nodule	27 (22.7%)	49 (67.1%)		40 (48.8%)		15 (38.5%)	
CTR (%)	58 (42, 81)	90 (59, 100)	<0.001 *	69 (47, 100)	0.902	62 (51, 77)	0.208
DLP (mm)	2.5 (0.0, 5.3)	0.0 (0.0, 3.0)	<0.001 *	0.0 (0.0, 3.9)	0.257	2.8 (0.0, 5.6)	0.461
PL (mm)	0.0 (0.0, 7.6)	12.3 (0.0, 17.9)	<0.001 *	5.9 (0.0, 11.2)	0.908	0.0 (0.0, 8.8)	0.957
SPL (mm)	0.0 (0.0, 3.0)	8.8 (0.0, 14.5)	<0.001 *	0 (0.0, 9.3)	0.116	0.0 (0.0, 8.7)	0.179
Tumor–pleura relationship			<0.001 *		0.687		0.721
Pleural tags	70 (58.8%)	23 (31.5%)		47 (57.3%)		22 (56.4%)	
Pleural attachment	49 (41.2%)	50 (68.5%)		35 (42.7%)		17 (43.6%)	
Lobulation			0.855		0.512		0.516
No	24 (20.2%)	16 (21.9%)		14 (17.1%)		6 (15.4%)	
Yes	95 (79.8%)	57 (78.1%)		68 (82.9%)		33 (84.6%)	
Spiculation			0.581		0.123		0.829
No	93 (78.2%)	60 (82.2%)		72 (87.8%)		30 (76.9%)	
Yes	26 (21.8%)	13 (17.8%)		10 (12.2%)		9 (23.1%)	
Air bronchogram			0.167		0.406		0.206
No	71 (59.7%)	51 (69.9%)		57 (69.5%)		20 (51.3%)	
Yes	48 (40.3%)	22 (30.1%)		25 (30.5%)		19 (48.7%)	
Vascular convergence sign			0.425		0.205		0.703
No	84 (70.6%)	51 (64.4%)		51 (62.2%)		26 (66.7%)	
Yes	35 (29.4%)	22 (35.6%)		31 (37.8%)		13 (33.3%)	

Quantitative features are medians, with interquartile ranges in parentheses, while others are the number of nodules. *p* ^a^ values for comparisons between VPI-negative and VPI-positive nodules within the training set. *p* ^b^ values for comparisons between the validation set and the training set (first column) and between the external test set and the training set (second column), regardless of VPI status. * *p* value < 0.05. CTR, consolidation-to-tumor ratio; DLP, minimum distance between lesion and pleura; PL, lesion-pleural contact length; SPL, solid component–pleural contact length.

**Table 2:** Multivariable logistic regression analysis of the radiological, radiomic, and habitat features in the training set.

Models	Features	Odds Ratio	95% CI	*p*
Radiological model	Solid component diameter	1.175	1.110–1.245	<0.001
	SPL	1.065	1.003–1.129	0.038
Radiomic model	GLCM_ClusterShade	0.355	0.189–0.668	0.001
	GLDM_LargeDependenceEmphasis	1.862	1.018–3.404	0.044
	GLDM_LargeDependenceLowGrayLevelEmphasis	0.155	0.031–0.787	0.025
Habitat model	Habitat1_firstorder_Skewness	0.494	0.300–0.812	0.005
	Habitat1_GLDM_SmallDependenceEmphasis	1.655	1.057–2.593	0.028
	Habitat2_NGTDM_Busyness	0.291	0.169–0.500	<0.001
	Habitat3_GLCM_ClusterProminence	0.438	0.194–0.991	0.047
	Habitat3_NGTDM_Contrast	0.491	0.276–0.874	0.016

CI, confidence intervals. SPL, solid component-pleural contact length; GLCM, gray level co-occurrence matrix; GLDM, gray level dependence matrix; NGTDM, neighboring gray tone difference matrix.

**Table 3:** Diagnostic performance of the radiological, radiomic, and habitat models.

Models	AUC	Sensitivity	Specificity	PPV	NPV
Training set					
Habitat model	0.914 (0.864–0.949)	64/73 [87.7%]	107/119 [89.9%]	64/76 [84.2%]	107/116 [92.2%]
Radiomic model	0.858 (0.800–0.904)	61/73 [83.6%]	100/119 [84.0%]	61/80 [76.2%]	100/112 [89.3%]
Radiological model	0.803 (0.739–0.856)	58/73 [79.5%]	90/119 [75.6%]	58/87 [66.7%]	90/105 [85.7%]
Validation set					
Habitat model	0.893 (0.806–0.951)	29/34 [85.3%]	42/48 [87.5%]	29/35 [82.9%]	42/47 [89.4%]
Radiomic model	0.833 (0.735–0.907)	26/34 [76.5%]	40/48 [83.3%]	26/34 [76.5%]	40/48 [83.3%]
Radiological model	0.746 (0.638–0.836)	24/34 [70.6%]	35/48 [72.9%]	24/37 [64.9%]	35/45 [77.8%]
External test set					
Habitat model	0.908 (0.772–0.977)	13/15 [86.7%]	20/24 [83.3%]	13/17 [76.5%]	20/22 [90.9%]
Radiomic model	0.772 (0.610–0.891)	11/15 [73.3%]	19/24 [79.2%]	11/16 [68.8%]	19/23 [82.6%]
Radiological model	0.624 (0.454–0.773)	10/15 [66.7%]	17/24 [70.8%]	10/17 [58.8%]	17/22 [77.3%]

Data in the parentheses are 95% confidence intervals. AUC, area under curve; PPV, positive predictive value; NPV, negative predictive value. The wide confidence intervals for the external test set reflect the limited sample size; point estimates should be interpreted with caution.

**Table 4:** Comparisons of the areas under curve of the radiological, radiomic, and habitat models through the Delong test.

Pairwise Comparison	Training Set		Validation Set		External Test Set	
	Z	*p*	Z	*p*	Z	*p*
Habitat model vs. radiomic model	2.297	0.022	2.032	0.042	2.432	0.015
Habitat model vs. radiological model	4.066	<0.001	3.373	<0.001	3.672	<0.001
Radiomic model vs. radiological model	2.445	0.015	2.177	0.030	2.129	0.033

## Data Availability

The data presented in this study are available on request from the corresponding author. The data are not publicly available due to the institutional privacy policies.

## Supplementary Materials

The following supporting information can be downloaded at: https://www.mdpi.com/article/10.3390/diagnostics16081191/s1, Supplementary Table S1: The acquisition and reconstruction parameters in three centers; Supplementary Table S2: Overview of radiomic features; Supplementary Table S3: Univariable logistic regression analysis of the radiological, radiomic, and habitat features in the training set; Supplementary Table S4: The mean and standard deviation of the radiomic and habitat features in original scale; Supplementary Table S5: The variance inflation factor of the selected features for the radiological, radiomic, and habitat models in the training set; Supplementary Table S6: STARD Checklist; Supplementary Table S7: The six key domains of the radiomics quality score; Supplementary Figure S1: Feature selection process of the radiomic and habitat features after the least absolute shrinkage and selection operator; Supplementary Figure S2: The corresponding confusion matrices of the habitat, radiomic, and radiological models; Supplementary Figure S3: The calibration curves of the habitat, radiomic, and radiological models; Supplementary Figure S4: The decision curve analysis of the habitat, radiomic, and radiological models in the combined validation and external test sets; Supplementary Method: Detailed Implementation of ComBat Harmonization. Supplementary Figure S5: The proportion of variance in features explained by batch (R^2^) before and after ComBat. Software Manual: Image Analysis Pipeline for Visceral Pleural Invasion Prediction on Low-Dose Computed Tomography.

## Abbreviations

The following abbreviations are used in this manuscript:

LDCT	Low-dose computed tomography
AUC	Area under the curve
CTR	Consolidation-to-tumor ratio
DCA	Decision curve analysis
DLP	Distance from lesion to the pleura
IQR	Interquartile range
ICC	Intra-class correlation coefficient
LASSO	Least absolute shrinkage and selection operator
NPV	Negative predictive value
PL	Pleural contact length
PPV	Positive predictive value
PCA	Principal component analysis
ROC	Receiver operating characteristic
SLIC	Simple linear iterative clustering
SPL	Solid tumor-pleural contact length
STARD	Standards for reporting diagnostic accuracy
VPI	Visceral pleural invasion
VOI	Volume of interest

## References

[1] de Koning H.J., van der Aalst C.M., de Jong P.A., Scholten E.T., Nackaerts K., Heuvelmans M.A., Lammers J.J., Weenink C., Yousaf-Khan U., Horeweg N. (2020). Reduced Lung-Cancer Mortality with Volume CT Screening in a Randomized Trial. N. Engl. J. Med..

[2] DeLong E.R., DeLong D.M., Clarke-Pearson D.L. (1988). Comparing the areas under two or more correlated receiver operating characteristic curves: A nonparametric approach. Biometrics.

[3] Peng H., Long F., Ding C. (2005). Feature selection based on mutual information criteria of max-dependency, max-relevance, and min-redundancy. IEEE Trans. Pattern Anal. Mach. Intell..

[4] Heidinger B.H., Schwarz-Nemec U., Anderson K.R., de Margerie-Mellon C., Monteiro Filho A.C., Chen Y., Mayerhoefer M.E., VanderLaan P.A., Bankier A.A. (2019). Visceral Pleural Invasion in Pulmonary Adenocarcinoma: Differences in CT Patterns between Solid and Subsolid Cancers. Radiol. Cardiothorac. Imaging.

[5] Nicholson A.G., Tsao M.S., Beasley M.B., Borczuk A.C., Brambilla E., Cooper W.A., Dacic S., Jain D., Kerr K.M., Lantuejoul S. (2022). The 2021 WHO Classification of Lung Tumors: Impact of Advances Since 2015. J. Thorac. Oncol..

[6] Gorai A., Sakao Y., Kuroda H., Uehara H., Mun M., Ishikawa Y., Nakagawa K., Masuda M., Okumura S. (2015). The clinicopathological features associated with skip N2 metastases in patients with clinical stage IA non-small-cell lung cancer. Eur. J. Cardio-Thorac. Surg..

[7] Yang H., Mei T. (2022). Prognostic significance of visceral pleural invasion in patients with surgically resected small-cell lung cancer: A population-based study. Jpn. J. Clin. Oncol..

[8] Wang C., Wu Y., Shao J., Liu D., Li W. (2020). Clinicopathological variables influencing overall survival, recurrence and post-recurrence survival in resected stage I non-small-cell lung cancer. BMC Cancer.

[9] Hsu J.S., Jaw T.S., Yang C.J., Lin S.F., Shih M.P., Chou S.H., Chong I.W., Lin M.Y., Chiang I.C. (2017). Convex border of peripheral non-small cell lung cancer on CT images as a potential indicator of pleural invasion. Medicine.

[10] Hsu J.S., Han I.T., Tsai T.H., Lin S.F., Jaw T.S., Liu G.C., Chou S.H., Chong I.W., Chen C.Y. (2016). Pleural Tags on CT Scans to Predict Visceral Pleural Invasion of Non-Small Cell Lung Cancer That Does Not Abut the Pleura. Radiology.

[11] Meng Y., Gao J., Wu C., Xie M., Ma X., Zang X., Song J., Zhou M., Guo S., Huang Y. (2022). The prognosis of different types of pleural tags based on radiologic-pathologic comparison. BMC Cancer.

[12] Onoda H., Higashi M., Murakami T., Tao H., Yokoyama S., Kunihiro Y., Kawano R., Tanabe M., Tanaka N., Matsumoto T. (2021). Correlation between pleural tags on CT and visceral pleural invasion of peripheral lung cancer that does not appear touching the pleural surface. Eur. Radiol..

[13] Huang S., Xu F., Zhu W., Xie D., Lou K., Huang D., Hu H. (2023). Multi-dimensional radiomics analysis to predict visceral pleural invasion in lung adenocarcinoma of ≤3 cm maximum diameter. Clin. Radiol..

[14] Wu Y.J., Wu F.Z., Yang S.C., Tang E.K., Liang C.H. (2022). Radiomics in Early Lung Cancer Diagnosis: From Diagnosis to Clinical Decision Support and Education. Diagnostics.

[15] Wu Y.J., Liu Y.C., Liao C.Y., Tang E.K., Wu F.Z. (2021). A comparative study to evaluate CT-based semantic and radiomic features in preoperative diagnosis of invasive pulmonary adenocarcinomas manifesting as subsolid nodules. Sci. Rep..

[16] Hausser J., Alon U. (2020). Tumour heterogeneity and the evolutionary trade-offs of cancer. Nat. Rev. Cancer.

[17] Huang S., Liang X., Lou K., Zhou J., Wang J., Xu G., Wu S., Hu H., Dong H. (2025). Comparing Habitat, Radiomics, and Fusion Models for Predicting Micropapillary/Solid Components in Stage I Lung Adenocarcinoma. Acad. Radiol..

[18] Xu P., Yao F., Xu Y., Yu H., Li W., Zhi S., Peng X. (2025). Habitat Radiomics and Deep Learning Features Based on CT for Predicting Lymphovascular Invasion in T1-stage Lung Adenocarcinoma: A Multicenter Study. Acad. Radiol..

[19] Shang Y., Zeng Y., Luo S., Wang Y., Yao J., Li M., Li X., Kui X., Wu H., Fan K. (2024). Habitat Imaging With Tumoral and Peritumoral Radiomics for Prediction of Lung Adenocarcinoma Invasiveness on Preoperative Chest CT: A Multicenter Study. AJR Am. J. Roentgenol..

[20] Mackin D., Fave X., Zhang L., Fried D., Yang J., Taylor B., Rodriguez-Rivera E., Dodge C., Jones A.K., Court L. (2015). Measuring Computed Tomography Scanner Variability of Radiomics Features. Investig. Radiol..

[21] Li Y., Long Y., Zheng Y., Liang J., Lin W., Qing H., Zhou P., Liu J. (2025). Ternary-Classification Habitat Model for Invasiveness and Grade of Lung Adenocarcinoma Presenting as a Subsolid Nodule on Low-Dose Chest CT: A Multicenter Study. AJR Am. J. Roentgenol..

[22] Zhao Q., Wang J.W., Yang L., Xue L.Y., Lu W.W. (2019). CT diagnosis of pleural and stromal invasion in malignant subpleural pure ground-glass nodules: An exploratory study. Eur. Radiol..

[23] Liu Y.C., Liang C.H., Wu Y.J., Chen C.S., Tang E.K., Wu F.Z. (2023). Managing Persistent Subsolid Nodules in Lung Cancer: Education, Decision Making, and Impact of Interval Growth Patterns. Diagnostics.

[24] Bossuyt P.M., Reitsma J.B., Bruns D.E., Gatsonis C.A., Glasziou P.P., Irwig L., Lijmer J.G., Moher D., Rennie D., de Vet H.C. (2015). STARD 2015: An updated list of essential items for reporting diagnostic accuracy studies. BMJ.

[25] Asamura H., Nishimura K.K., Giroux D.J., Chansky K., Hoering A., Rusch V., Rami-Porta R. (2023). IASLC Lung Cancer Staging Project: The New Database to Inform Revisions in the Ninth Edition of the TNM Classification of Lung Cancer. J. Thorac. Oncol..

[26] Zwanenburg A., Vallières M., Abdalah M.A., Aerts H., Andrearczyk V., Apte A., Ashrafinia S., Bakas S., Beukinga R.J., Boellaard R. (2020). The Image Biomarker Standardization Initiative: Standardized Quantitative Radiomics for High-Throughput Image-based Phenotyping. Radiology.

[27] Fortin J.P., Cullen N., Sheline Y.I., Taylor W.D., Aselcioglu I., Cook P.A., Adams P., Cooper C., Fava M., McGrath P.J. (2018). Harmonization of cortical thickness measurements across scanners and sites. Neuroimage.

[28] Orlhac F., Frouin F., Nioche C., Ayache N., Buvat I. (2019). Validation of A Method to Compensate Multicenter Effects Affecting CT Radiomics. Radiology.

[29] Suzuki K., Koike T., Asakawa T., Kusumoto M., Asamura H., Nagai K., Tada H., Mitsudomi T., Tsuboi M., Shibata T. (2011). A prospective radiological study of thin-section computed tomography to predict pathological noninvasiveness in peripheral clinical IA lung cancer (Japan Clinical Oncology Group 0201). J. Thorac. Oncol..

[30] Wang Q., Zhou X., Wang C., Liu Z., Huang J., Zhou Y., Li C., Zhuang H., Cheng J.Z. (2019). WGAN-Based Synthetic Minority Over-Sampling Technique: Improving Semantic Fine-Grained Classification for Lung Nodules in CT Images. IEEE Access.

[31] Mu G., Chen Y., Wu D., Zhan Y., Zhou X.S., Gao Y. (2019). Relu Cascade of Feature Pyramid Networks for CT Pulmonary Nodule Detection. Machine Learning in Medical Imaging. MLMI 2019.

[32] Li Y., Liu J., Yang X., Wang A., Zang C., Wang L., He C., Lin L., Qing H., Ren J. (2023). An ordinal radiomic model to predict the differentiation grade of invasive non-mucinous pulmonary adenocarcinoma based on low-dose computed tomography in lung cancer screening. Eur. Radiol..

[33] Tibshirani R. (2018). Regression Shrinkage and Selection Via the Lasso. J. R. Stat. Soc. Ser. B (Methodol.).

[34] Achanta R., Shaji A., Smith K., Lucchi A., Fua P., Süsstrunk S. (2012). SLIC superpixels compared to state-of-the-art superpixel methods. IEEE Trans. Pattern Anal. Mach. Intell..

[35] Zhang Y., Wang S., Song M., Sheng R., Geng Z., Zhang W., Zeng M. (2025). MRI-based Intra- and Peritumoral Heterogeneity in Hepatocellular Carcinoma for Microvascular Invasion Prediction and Prognostic Risk Stratification. Radiol. Imaging Cancer.

[36] Sujit S.J., Aminu M., Karpinets T.V., Chen P., Saad M.B., Salehjahromi M., Boom J.D., Qayati M., George J.M., Allen H. (2024). Enhancing NSCLC recurrence prediction with PET/CT habitat imaging, ctDNA, and integrative radiogenomics-blood insights. Nat. Commun..

[37] Hotelling H. (1933). Analysis of a complex of statistical variables into principal components. J. Educ. Psychol..

[38] Stekhoven D.J., Bühlmann P. (2012). MissForest—Non-parametric missing value imputation for mixed-type data. Bioinformatics.

[39] Yu Y., Huang R., Wang P., Wang S., Ling X., Zhang P., Yu J., Wang J., Xiao J., Wang Z. (2020). Sublobectomy versus lobectomy for long-term survival outcomes of early-stage non-small cell lung cancer with a tumor size ≤2 cm accompanied by visceral pleural invasion: A SEER population-based study. J. Thorac. Dis..

[40] Huang R., Zhao C., Yang J., Lu B., Dai Y., Lin M., Zhao X., Huang H., Pan X., Lu L. (2025). Nomogram based on radiomics and CT features for predicting visceral pleural invasion of invasive adenocarcinoma ≤ 2 cm: A multicenter study. Eur. J. Radiol..

[41] Shi Z., Huang X., Cheng Z., Xu Z., Lin H., Liu C., Chen X., Liu C., Liang C., Lu C. (2023). MRI-based Quantification of Intratumoral Heterogeneity for Predicting Treatment Response to Neoadjuvant Chemotherapy in Breast Cancer. Radiology.

[42] Yang Y.C., Wu J.J., Shi F., Ren Q.G., Jiang Q.J., Guan S., Tang X.Q., Meng X.S. (2025). Sub-regional Radiomics Analysis for Predicting Metastasis Risk in Clear Cell Renal Cell Carcinoma: A Multicenter Retrospective Study. Acad. Radiol..

[43] Ganeshan B., Miles K.A. (2013). Quantifying tumour heterogeneity with CT. Cancer Imaging.

[44] van Griethuysen J.J.M., Fedorov A., Parmar C., Hosny A., Aucoin N., Narayan V., Beets-Tan R.G.H., Fillion-Robin J.C., Pieper S., Aerts H. (2017). Computational Radiomics System to Decode the Radiographic Phenotype. Cancer Res..

[45] Shimizu K., Yoshida J., Nagai K., Nishimura M., Ishii G., Morishita Y., Nishiwaki Y. (2005). Visceral pleural invasion is an invasive and aggressive indicator of non-small cell lung cancer. J. Thorac. Cardiovasc. Surg..

[46] Berenguer R., Pastor-Juan M.D.R., Canales-Vázquez J., Castro-García M., Villas M.V., Mansilla Legorburo F., Sabater S. (2018). Radiomics of CT Features May Be Nonreproducible and Redundant: Influence of CT Acquisition Parameters. Radiology.

