# Supplementary material for: Habitat-Based Radiomics for Predicting Visceral Pleural Invasion in Subpleural Nodules with Solid Component on Low-Dose CT: A Multicenter Study
Source: Diagnostics (Basel). 2026 Apr 16;16(8):1191. doi: 10.3390/diagnostics16081191 (PMC13114590; doi:10.3390/diagnostics16081191)
Supplement: Supplementary file 1 [file diagnostics-16-01191-s001.zip › diagnostics-4222974-supplementary.pdf]

Supplementary Material

Supplementary Tables

Supplementary Table 1. The acquisition and reconstruction parameters in three centers

Parameters	Center 1	Center 2		Center 3	
Scanner	SOMATOM Definition Flash	SOMATOM Force	Philips Brilliance Big Bore	SOMATOM Definition Flash	UIH uCT 780
Tube voltage (kVp)	100	80 or 90 or 100	100	100	70 or 100
Tube current (mAs)	10 or 20 or 30	40 to 58	30 to 73	46 to 75	36 to 85
Pitch	1	1.2 or 2	0.938 or 1.063	1.2 or 1.5	1.213
Collimation (mm)	64 × 0.6	96 × 0.6	16 × 0.75	64 × 0.6	80 × 0.5
Rotation time (s)	0.33	0.25 or 0.5	0.5	0.285 or 0.33 or 0.5	0.5
Field of view (mm)	350 × 350	350 × 350	350 × 350	350 × 350	350 × 350
Reconstruction Kernel	I30f\5	Br40d\3	A	I30f\3	B_SOFE_B
Slice thickness (mm)	0.5	0.75	1	1	1
Slice increment (mm)	0.5	0.75	1	1	1
Matrix	512 × 512	512 × 512	512 × 512	512 × 512	512 × 512
Voxel size (mm^3)	0.684 × 0.684 × 0.5	0.684 × 0.684 × 0.75	0.684 × 0.684 × 1	0.684 × 0.684 × 1	0.684 × 0.684 × 1

Supplementary Table 2. Overview of radiomic features

Classification	Features	Counts
Shape	Elongation, Flatness, Least axis length, Major axis length, Maximum 2D diameter (column), Maximum 2D diameter (row), Maximum 2D diameter (slice), Maximum 3D diameter, Mesh volume, Minor axis length, Sphericity, Surface area, Surface area to volume ratio, Voxel volume	14
First order	10 Percentile, 90 Percentile, Energy, Entropy, Interquartile range, Kurtosis, Maximum, Mean absolute deviation, Mean, Median, Minimum, Range, Robust mean absolute deviation, Root mean squared, Skewness, Total energy, Uniformity, Variance	18
Texture		
GLCM	Autocorrelation, Cluster prominence, Cluster shade, Cluster tendency, Contrast, Correlation, Difference average, Difference entropy, Difference variance, Inverse difference normalized (Idn), Inverse difference (Id), Inverse difference moment normalized (Idmn), Inverse difference moment (Idm), Informational measure of correlation 1 (Imc1), Informational measure of correlation 2 (Imc2), Inverse variance, Joint average, Joint energy, Joint entropy, Maximum probability, Maximal Correlation Coefficient, Sum entropy, Sum Squares.	23
GLRLM	Gray level non-uniformity (GLN), Gray level non-uniformity normalized (GLNN), Gray level variance (GLV), High gray level run emphasis (HGLRE), Long run emphasis (LRE), Long run high gray level emphasis (LRHGLE), Long run low gray level emphasis (LRLGLE), Low gray level run emphasis (LGLRE), Run entropy (RE), Run length non-uniformity (RLN), Run length non-uniformity normalized (RLNN), Run percentage (RP), Run variance (RV), Short run emphasis (SRE), Short run high gray level emphasis (SRHGLE), Short run low gray level emphasis (SRLGLE)	16
GLSZM	Gray level non-uniformity (GLN), Gray level non-uniformity normalized (GLNN), Gray level variance (GLV), High gray level zone emphasis (HGLZE), Large area emphasis (LAE), Large area high gray level emphasis (LAHGLE), Large area low gray level emphasis (LALGLE), Low gray level zone emphasis (LGLZE), Size zone non-uniformity (SZN), Size zone non-uniformity normalized (SZNN), Small area	16

	emphasis (SAE), Small area high gray level emphasis (SAHGLE), Small area low gray level emphasis (SALGLE), Zone entropy (ZE), Zone percentage (ZP), Zone variance (ZV)	
GLDM	Dependence entropy (DE), Dependence non-uniformity (DN), Dependence non-uniformity normalized (DNN), Dependence variance (DV), Gray level non-uniformity (GLN), Gray level variance (GLV), High gray level emphasis (HGLE), Large dependence emphasis (LDE), Large dependence high gray level emphasis (LDHGLE), Large dependence low gray level emphasis (LDLGLE), Low gray level emphasis (LGLE), Small dependence emphasis (SDE), Small dependence high gray level emphasis (SDHGLE), Small dependence low gray level emphasis (SDLGLE)	14
NGTDM	Busyness, Coarseness, Complexity, Contrast, Strength	5

GLCM, gray level co-occurrence matrix; GLRLM, gray level run length matrix; GLSZM, gray level size zone matrix; GLDM, gray level dependence matrix; NGTDM, neighbouring gray tone difference matrix

Supplementary Table 3. Univariable logistic regression analysis of the radiological, radiomic, and habitat features in the training set

Models	Features	Odds ratio	95% CI	*P*
Radiological model	Maximal diameter	1.073	1.012–1.137	0.019*
	Solid component diameter	1.192	1.127–1.260	<0.001*
	CTR	1.045	1.030–1.060	<0.001*
	DLP	0.997	0.922–1.077	0.933
	PL	1.030	0.990–1.072	0.145
	SPL	1.108	1.053–1.165	<0.001*
	Nodule type			<0.001*
	Part solid nodule	Reference	/	/
	Solid nodule	6.957	3.632–13.326	<0.001*
	Tumor–pleura relationship			
	Pleural tags	Reference	/	/
	Pleural attachment	1.032	0.576–1.850	0.915
Radiomic model	Firstorder_Maximum	2.920	1.805–4.723	<0.001*
	GLCM_ClusterShade	0.238	0.153–0.370	<0.001*
	GLDM_LargeDependenceEmphasis	3.613	2.302–5.673	<0.001*
	GLDM_LargeDependenceLowGrayLevelEmphasis	0.065	0.006–0.667	0.021*
	GLRLM_GrayLevelNonUniformityNormalized	1.877	1.363–2.583	<0.001*
	NGTDM_Strength	0.611	0.407–0.917	0.017
Habitat model	Habitat1_Firstorder_Skewness	0.276	0.182–0.420	<0.001*
	Habitat1_GLDM_SmallDependenceEmphasis	1.973	1.386–2.808	<0.001*
	Habitat2_GLCM_ClusterShade	0.509	0.349–0.743	<0.001*
	Habitat2_GLRLM_GrayLevelNonUniformity	1.770	1.291–2.428	<0.001*
	Habitat2_GLSZM_SmallAreaEmphasis	2.032	1.421–2.905	<0.001*
	Habitat2_NGTDM_Busyness	0.459	0.316–0.667	<0.001*
	Habitat3_Firstorder_RootMeanSquared	0.472	0.331–0.673	<0.001*
	Habitat3_GLCM_ClusterProminence	0.333	0.227–0.488	<0.001*
	Habitat3_NGTDM_Contrast	0.291	0.183–0.462	<0.001*

CI, confidence interval; CTR, consolidation-to-tumor ratio; DLP, minimum distance between lesion and pleura; PL, lesion-pleural contact length; SPL, solid component-pleural contact length; GLCM, gray level co-occurrence matrix; GLRLM, gray level run length matrix; GLSZM, gray level size zone matrix; GLDM, gray level dependence matrix; NGTDM, neighbouring gray tone difference matrix. * $P < 0.05$.

Supplementary Table 4. The mean and standard deviation of the radiomic and habitat features in original scale

Models	Features	Mean	SD
Radiomic model	GLCM_ClusterShade	−1221.500	2808.169
	GLDM_LargeDependenceEmphasis	25.937	23.045
	GLDM_LargeDependenceLowGrayLevelEmphasis	0.147	0.430
Habitat model	Habitat1_Firstorder_Skewness	−1.783	0.801
	Habitat1_GLDM_SmallDependenceEmphasis	0.492	0.115
	Habitat2_NGTDM_Busyness	0.532	0.146
	Habitat3_GLCM_ClusterProminence	38133.298	34472.729
	Habitat3_NGTDM_Contrast	0.444	0.168

GLCM, gray level co-occurrence matrix; GLDM, gray level dependence matrix; NGTDM, neighbouring gray tone difference matrix.

Supplementary Table 5. The variance inflation factor of the selected features for the radiological, radiomic, and habitat models in the training set

Models	Features	VIF
Radiological model	Solid component diameter	1.146
	SPL	1.146
Radiomic model	GLCM_ClusterShade	1.706
	GLDM_LargeDependenceEmphasis	1.679
	GLDM_LargeDependenceLowGrayLevelEmphasis	1.035
Habitat model	Habitat1_Firstorder_Skewness	1.329
	Habitat1_GLDM_SmallDependenceEmphasis	1.103
	Habitat2_NGTDM_Busyness	1.396
	Habitat3_GLCM_ClusterProminence	1.361
	Habitat3_NGTDM_Contrast	1.029

SPL, solid component-pleural contact length; GLCM, gray level co-occurrence matrix; GLDM, gray level dependence matrix; NGTDM, neighbouring gray tone difference matrix.

Supplementary Table 6. STARD Checklist

Section & Topic	No	Item	Reported on page #
**TITLE OR ABSTRACT**			
	**1**	Identification as a study of diagnostic accuracy using at least one measure of accuracy (such as sensitivity, specificity, predictive values, or AUC)	Page 1
**ABSTRACT**			
	**2**	Structured summary of study design, methods, results, and conclusions (for specific guidance, see STARD for Abstracts)	Page 1
**INTRODUCTION**			
	**3**	Scientific and clinical background, including the intended use and clinical role of the index test	Page 2
	**4**	Study objectives and hypotheses	Page 2
**METHODS**			
Study design	**5**	Whether data collection was planned before the index test and reference standard were performed (prospective study) or after (retrospective study)	Page 3
Participants	**6**	Eligibility criteria	Page 3
	**7**	On what basis potentially eligible participants were identified (such as symptoms, results from previous tests, inclusion in registry)	Page 3
	**8**	Where and when potentially eligible participants were identified (setting, location and dates)	Page 3
	**9**	Whether participants formed a consecutive, random or convenience series	Fig.1
Test methods	**10a**	Index test, in sufficient detail to allow replication	Page 4-6
	**10b**	Reference standard, in sufficient detail to allow replication	Page 3
	**11**	Rationale for choosing the reference standard (if alternatives exist)	Page 2
	**12a**	Definition of and rationale for test positivity cut-offs or result categories of the index test, distinguishing pre-specified from exploratory	Page 6
	**12b**	Definition of and rationale for test positivity cut-offs or result categories of the reference	Page 3

		standard, distinguishing pre-specified from exploratory	
	**13a**	Whether clinical information and reference standard results were available to the performers/readers of the index test	Page 4
	**13b**	Whether clinical information and index test results were available to the assessors of the reference standard	N/A
Analysis	**14**	Methods for estimating or comparing measures of diagnostic accuracy	Page 6
	**15**	How indeterminate index test or reference standard results were handled	N/A
	**16**	How missing data on the index test and reference standard were handled	N/A
	**17**	Any analyses of variability in diagnostic accuracy, distinguishing pre-specified from exploratory	Page 6
	**18**	Intended sample size and how it was determined	N/A
**RESULTS**			
Participants	**19**	Flow of participants, using a diagram	Fig.1
	**20**	Baseline demographic and clinical characteristics of participants	Table 1
	**21a**	Distribution of severity of disease in those with the target condition	Table 1
	**21b**	Distribution of alternative diagnoses in those without the target condition	N/A
	**22**	Time interval and any clinical interventions between index test and reference standard	Page 3
Test results	**23**	Cross tabulation of the index test results (or their distribution) by the results of the reference standard	Supplementary Fig.2
	**24**	Estimates of diagnostic accuracy and their precision (such as 95% confidence intervals)	Table 3
	**25**	Any adverse events from performing the index test or the reference standard	N/A
**DISCUSSION**			
	**26**	Study limitations, including sources of potential bias, statistical uncertainty, and generalisability	Page 13
	**27**	Implications for practice, including the intended use and clinical role of the index test	Page 12-13
**OTHER INFORMATION**			
	**28**	Registration number and name of registry	N/A

	**29**	Where the full study protocol can be accessed	N/A
	**30**	Sources of funding and other support; role of funders	Page 14

Supplementary Table 7. The six key domains of the radiomics quality score

Domain			score	RQS criteria
1	Image protocol quality	Well-documented image protocols (for example, contrast, slice thickness, energy, etc.) and/or usage of public image protocols allow reproducibility/replicability	+ 1 (if protocols are well-documented)<br>+ 1 (if public protocol is used)	√
	Multiple segmentation s	Segmentation by different physicians/algorithms/software, perturbing segmentations by (random) noise, segmentation at different breathing cycles. Analyse feature robustness to segmentation variabilities	+ 1	√
	Phantom study on all scanners	Detect inter-scanner differences and vendor-dependent features. Analyse feature robustness to these sources of variability	+ 1	NA
	Imaging at multiple time points	Collect images of individuals at additional time points. Analyse feature robustness to temporal variabilities (for example, organ movement, organ expansion/ shrinkage)	+ 1	NA
2	Feature reduction or adjustment for multiple testing	Decreases the risk of overfitting. Overfitting is inevitable if the number of features exceeds the number of samples. Consider feature robustness when selecting features	- 3 (if neither measure is implemented)<br>+ 3 (if either measure is implemented)	√
	Validation	The validation is performed without retraining and without adaptation of the cut-off value, provides crucial information with regards to credible clinical performance	- 5 (if validation is missing)<br>+ 2 (if validation is based on a dataset from the same institute)<br>+ 3 (if validation is based on a dataset from another institute)<br>+ 4 (if validation is based on two datasets from two distinct institutes)<br>+ 4 (if the study validates a previously published signature)<br>+ 5 (if validation is based on three or more datasets from distinct institutes)	√ (+5)
3	Multivariable analysis with non-radiomics features	(for example, EGFR mutation) - is expected to provide a more holistic model. Permits correlating/inferencing between radiomics and non radiomics features	+1	√

	Detect and discuss biological correlates	Demonstration of phenotypic differences (possibly associated with underlying gene–protein expression patterns) deepens understanding of radiomics and biology	+1	√
	Comparison to 'gold standard'	Assess the extent to which the model agrees with/is superior to the current 'gold standard' method (for example, TNM-staging for survival prediction). This comparison shows the added value of radiomics	+2	√
	Potential clinical utility	Report on the current and potential application of the model in a clinical setting (for example, decision curve analysis).	+2	√
4	Cut-off analyses	Determine risk groups by either the median, a previously published cut-off or report a continuous risk variable. Reduces the risk of reporting overly optimistic results	+1	√
	Discrimination statistics	Report discrimination statistics (for example, C-statistic, ROC curve, AUC) and their statistical significance (for example, p-values, confidence intervals). One can also apply resampling method (for example, bootstrapping, cross-validation)	+ 1 (if a discrimination statistic and its statistical significance are reported) + 1 (if a resampling method technique is also applied)	√ (+2)
	Calibration statistics	Report calibration statistics (for example, Calibration-in-the-large/slope, calibration plots) and their statistical significance (for example, *P*-values, confidence intervals). One can also apply resampling method (for example, bootstrapping, cross-validation)	+ 1 (if a calibration statistic and its statistical significance are reported) + 1 (if a resampling method technique is also applied)	√ (+2)
5	Prospective study registered in a trial database	Provides the highest level of evidence supporting the clinical validity and usefulness of the radiomics biomarker	+ 7 (for prospective validation of a radiomics signature in an appropriate trial)	NA
	Cost-effectiveness analysis	Report on the cost-effectiveness of the clinical application (for example, QALYs generated)	+1	√

6	Open science and data	Make code and data publicly available. Open science facilitates knowledge transfer and reproducibility of the study	+ 1 (if scans are open source) + 1 (if region of interest segmentations are open source) + 1 (if code is open source) + 1 (if radiomics features are calculated on a set of representative ROIs and the calculated features and representative ROIs are open source)	√ (+2)

Total score = 24 (66.67%); NA, not applicable.

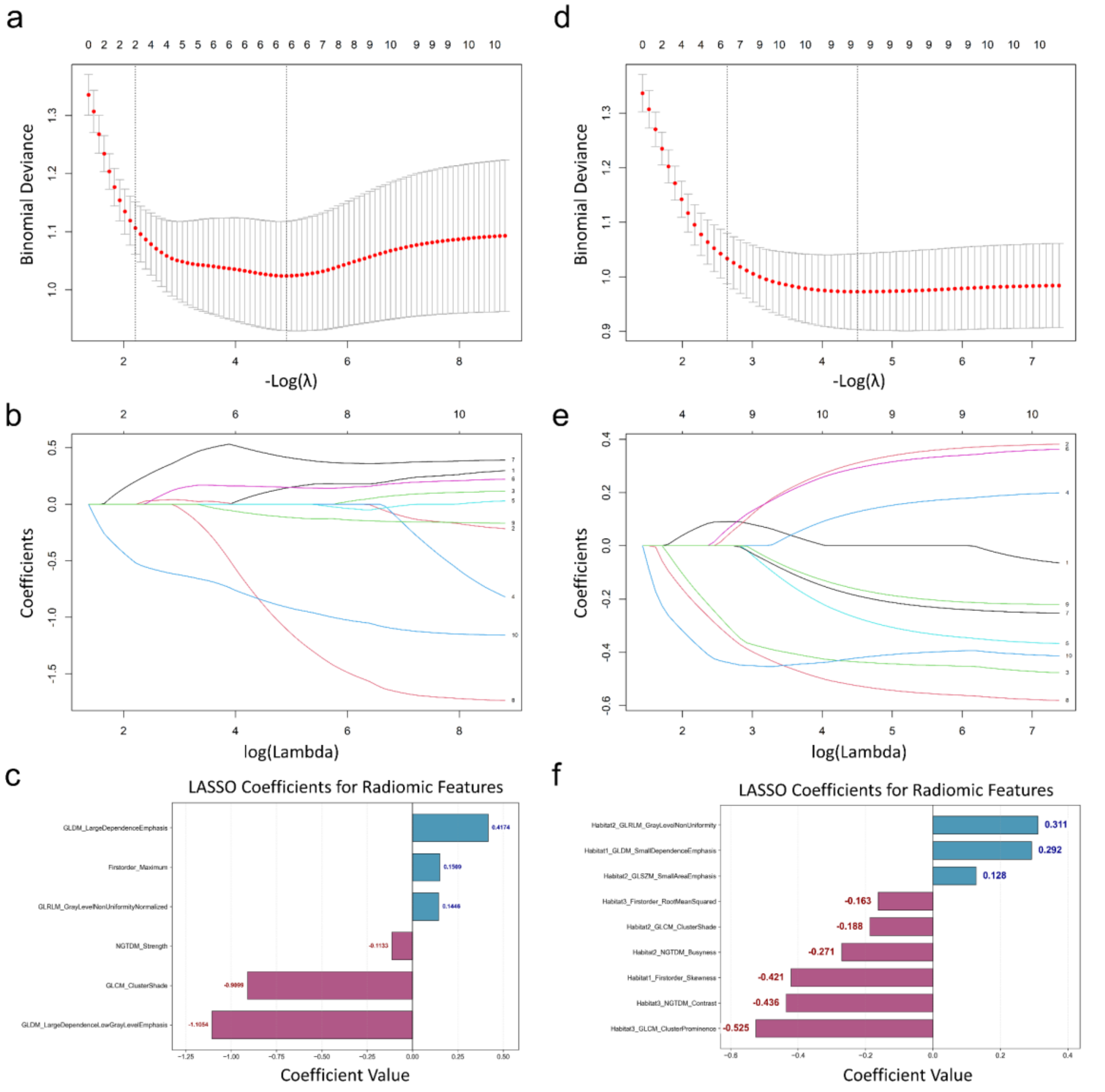

Supplementary Figure 1. Feature selection process of the radiomic and habitat features after the least absolute shrinkage and selection operator. **(a, b, c)** The radiomic features. **(d, e, f)** The habitat features. **(a, d)** Feature selection using the LASSO binary logistic regression model. The vertical dotted line on the left indicates the minimum criteria of λ using 10-fold cross-validation. **(b, e)** LASSO coefficient profiles of the features. **(c, f)** The coefficient value of each feature ranking by importance.

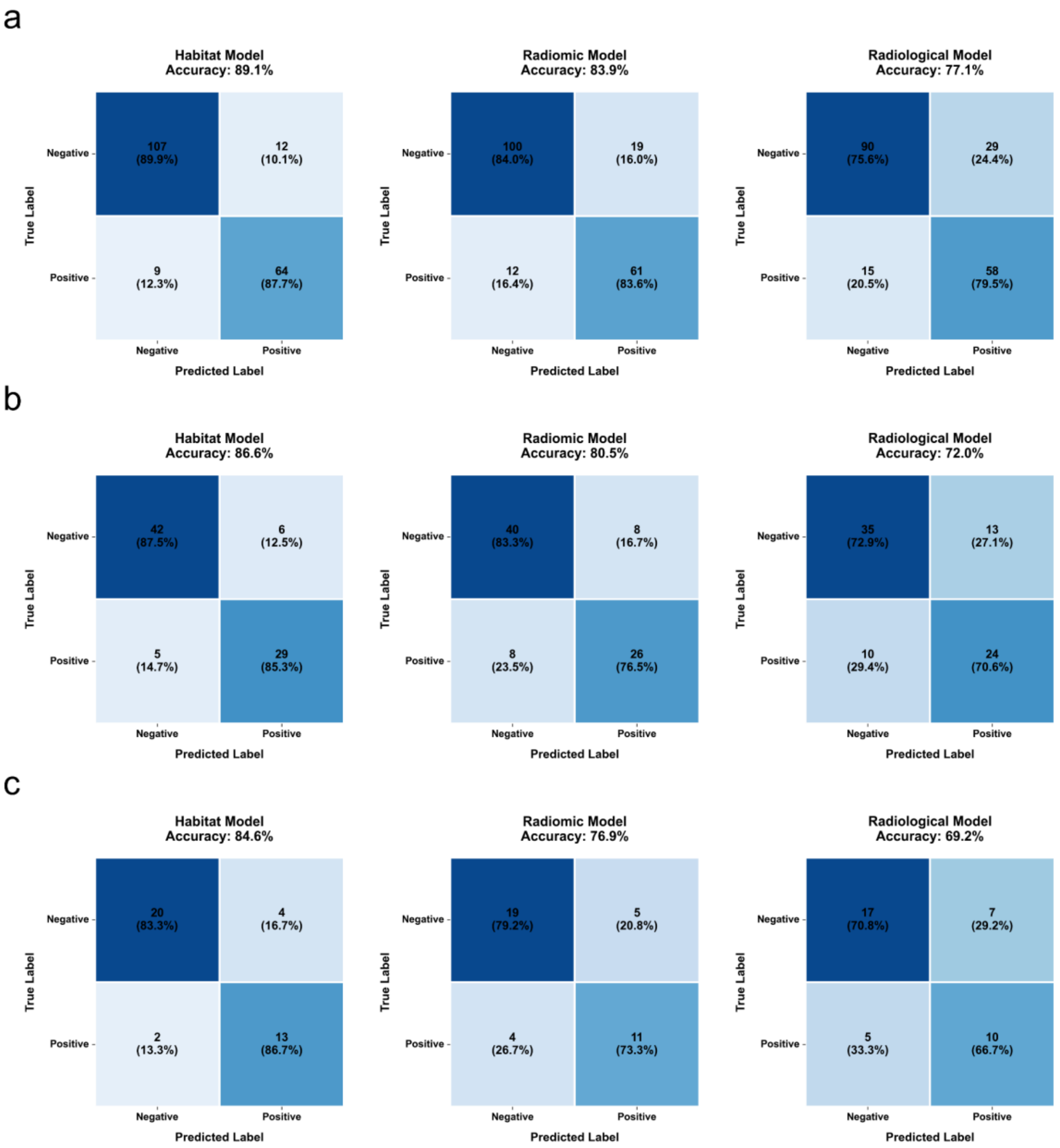

Supplementary Figure 2. The corresponding confusion matrices of the habitat, radiomic, and radiological models. **(a)** Training set. **(b)** Validation set. **(c)** External test set.

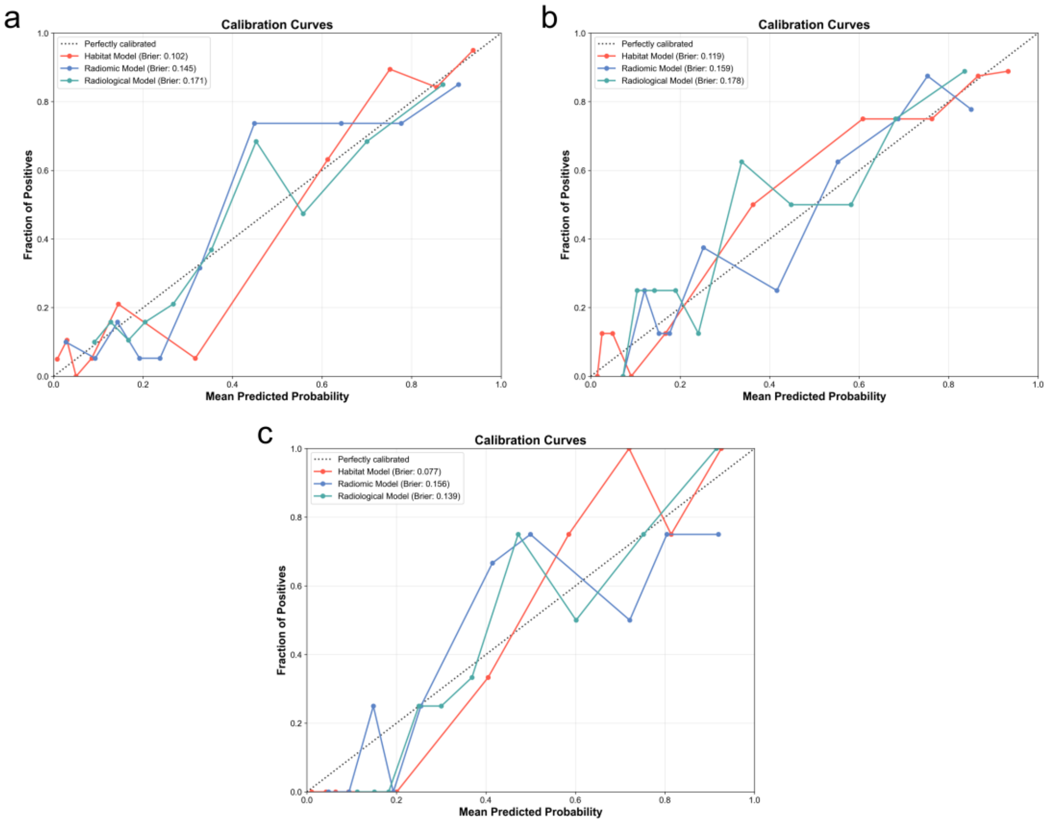

Supplementary Figure 3. The calibration curves of the habitat, radiomic, and radiological models. **(a)** Training set. **(b)** Validation set. **(c)** External test set.

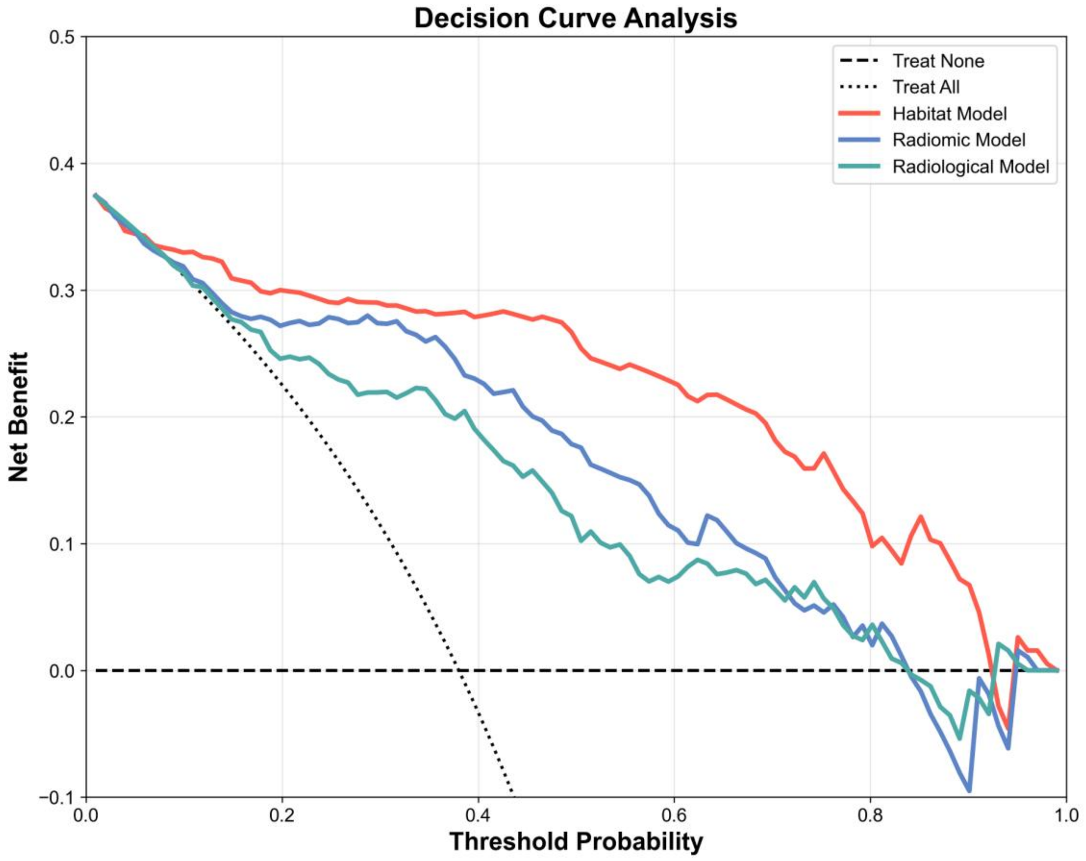

Supplementary Figure 4. The decision curve analysis of the habitat, radiomic, and radiological models in the combined validation and external test sets.

Supplementary Method: Detailed Implementation of ComBat Harmonization

1. Preprocessing steps prior to harmonization

To minimize inter-scanner variability and LDCT-related noise, the following preprocessing steps were applied to all images before feature extraction and harmonization:

1. Resampling: All LDCT images were resampled to an isotropic voxel size of 1 × 1 × 1 mm^3 using linear interpolation to standardize spatial resolution across centers.
2. Intensity discretization: Gray-level intensities were discretized into 25 fixed bins. This step suppresses high-frequency noise, reduces computational complexity, and improves the signal-to-noise ratio of radiomic features, which is particularly important for LDCT images.

2. Radiomic feature extraction

After preprocessing, a total of 106 radiomic features were extracted from each volume of interest (VOI) using PyRadiomics (version 3.0.1). These features comprised:

- 14 shape features
- 18 first-order features
- 23 Gray Level Co-occurrence Matrix (GLCM) features
- 16 Gray Level Run Length Matrix (GLRLM) features
- 16 Gray Level Size Zone Matrix (GLSZM) features
- 14 Gray Level Dependence Matrix (GLDM) features
- 5 Neighbouring Gray Tone Difference Matrix (NGTDM) features

For the habitat model, the same set of 106 features was extracted separately from each of the three habitat subregions, yielding 279 features (93 per habitat after removal of redundant shape features).

3. Definition of batch effects

Batch effects were defined based on the scanner type and acquisition protocol used at each participating center. Specifically, the three centers contributed data from five distinct CT scanners:

- Center 1: SOMATOM Definition Flash (one scanner)
- Center 2: SOMATOM Force and Philips Brilliance Big Bore (two scanners)
- Center 3: SOMATOM Definition Flash and UIH uCT 780 (two scanners)

Although some scanners were shared across centers (e.g., SOMATOM Definition Flash appeared in both Center 1 and Center 3), differences in acquisition parameters (tube voltage, tube current, rotation time, reconstruction kernel, slice thickness) across centers necessitated treating each center-scanner combination as a separate batch. Accordingly, five batches were defined in the ComBat model.

4. ComBat harmonization procedure

We applied the ComBat method implemented in the Python *neuroCombat* package, which was available at *https://github.com/Jfortin1/ComBatHarmonization*, following the empirical Bayes framework originally described by Fortin et al. [1] and adapted for CT radiomics by Orlhac et al. [2].

The model assumes that the observed feature value Yij for feature i in sample j can be decomposed as:

$$Y_{ij}=\alpha_i+X\beta_i+\gamma_{i,b(j)}+\delta_{i,b(j)}\epsilon_{ij}$$

where:

- α_i is the overall mean of feature i
- $X\beta_i$ represents biological covariates (here, VPI status) to be preserved
- $\gamma_{i,b(j)}$ is the additive batch effect for feature i in batch b containing sample j
- $\delta_{i,b(j)}$ is the multiplicative (scale) batch effect
- ϵ_{ij} is the random error

The empirical Bayes step pools information across features to obtain robust estimates of batch effect parameters, particularly when sample sizes per batch are limited.

5. Input and parameters

Data inputs for ComBat are:

- *A data matrix*. The data to harmonize. Rows are features (for instance voxels or brain regions) and columns are participants.
- *A batch id vector*. A vector (length should be equal to the number of columns in the data matrix) that specifies the id for the batch, site, or scanner to correct for. ComBat only accepts one batch vector. You should provide the smallest unit of the study that you believe introduces unwanted variation. For instance, for a study with 2 sites and 3 scanners (1 site with 1 scanner, 1 site with 2 scanners), the id for scanner should be used.
- *Biological variables*. Optional design matrix specifying biological covariates that should be protected for during the removal of scanner/site effects, such as disease status, age, gender, etc.

There are several alternative modes of running ComBat:

- parametric = FALSE: will instead use a non-parametric prior method in the empirical Bayes procedure (default uses parametric priors).
- eb = FALSE: will not run the empirical Bayes procedure, and therefore location and scale parameters are not shrunk towards common factors averaged across features. This is equivalent to running a location-and-scale correction method for each feature separately. This is particularly useful for debugging and method development.
- mean.only = TRUE: will only adjust the mean of the site effects across sites (default adjusts for mean and variance). This option is recommended for studies where the variances are expected to be different across sites due to the biology.

6. Key implementation details:

- Biological covariate: VPI status (positive vs. negative) was included as a covariate in the model to ensure that true biological differences were preserved while scanner-related effects were removed.
- Parameter estimation: The ComBat method was fitted exclusively on the training set (n = 192). The estimated batch effect parameters (additive and multiplicative corrections for each feature and batch) were saved and subsequently applied to the validation and

external test sets. This approach prevents data leakage and ensures unbiased performance evaluation.

- Features harmonized: All 106 radiomic features (for the whole-lesion radiomic model) and all 279 habitat-specific features (for the habitat model) were harmonized. No pre-selection of features was performed before ComBat, as the method is designed to handle high-dimensional data directly.

7. Validation of harmonization effectiveness

To quantitatively assess the effectiveness of harmonization, we computed the proportion of variance in each radiomic feature explained by batch (R^2) before and after ComBat, averaged across all features in the training set. The mean R^2 decreased from 1.17% before harmonization to 0.49% after harmonization, representing a 58.2% relative reduction. A paired *t*-test showed the reduction was statistically significant ($P < 0.001$). These results confirm that our ComBat pipeline effectively reduced inter-scanner variability in the training set.

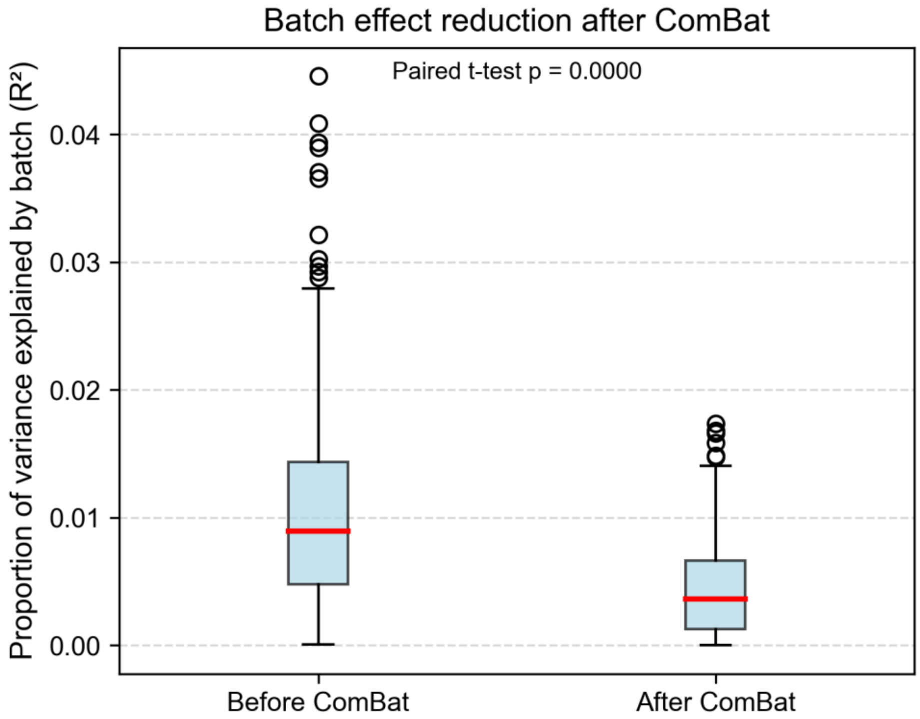

Supplementary Figure 5. The proportion of variance in features explained by batch (R^2) before and after ComBat.

Reference:

1. Fortin, J.P.; Cullen, N.; Sheline, Y.I.; Taylor, W.D.; Aselcioglu, I.; Cook, P.A.; Adams, P.; Cooper, C.; Fava, M.; McGrath, P.J.; et al. Harmonization of cortical thickness measurements across scanners and sites. *Neuroimage* **2018**, *167*, 104-120, doi:10.1016/j.neuroimage.2017.11.024.
2. Orlhac, F.; Frouin, F.; Nioche, C.; Ayache, N.; Buvat, I. Validation of A Method to Compensate Multicenter Effects Affecting CT Radiomics. *Radiology* **2019**, *291*, 53-59, doi:10.1148/radiol.2019182023.

Software Manual: Image Analysis Pipeline for Visceral Pleural Invasion Prediction on Low-Dose Computed Tomography

1. Software Overview

1.1 Software Introduction

This software is an automated tool specifically designed for analyzing low-dose computed tomography (LDCT) images, aiming to predict visceral pleural invasion (VPI) status in subpleural nodules with component detected during lung cancer screening. The software integrates advanced image preprocessing (e.g., resampling and normalization), 3D SLIC superpixel segmentation, habitat clustering, radiomic feature extraction per habitat, and logistic regression based prediction to provide quantitative support for clinical decision-making.

1.2 Key Features

- **Single Case Processing**: Analyze CT images and segmentation masks for individual patients
- **Batch Processing**: Automatically process multiple cases in a directory
- **Comprehensive Analysis Pipeline**: Includes five processing steps
- **Result Visualization**: Intuitive display of prediction results and feature values
- **Data Export**: Export results to CSV and Excel formats

1.3 Software Interface

The software features a tabbed graphical user interface with four main sections:

- **Tab 1: Input & Processing** - File selection and processing controls
- **Tab 2: Single Case Results** - Detailed results for individual cases
- **Tab 3: Batch Results** - Summary results for batch processing
- **Tab 4: About** - Software information and instructions

2. File Requirements and Format

2.1 Supported File Formats

- **CT Images**: .nii.gz, .nii
- **Segmentation Masks**: Same formats as CT images

2.2 File Naming Conventions

The software automatically matches CT and mask files. Supported naming patterns include:

CT File	Mask File	Example
name.nii.gz	name_mask.nii.gz	patient01.nii.gz ↔ patient01_mask.nii.gz
name.nii	name_seg.nii	patient02.nii ↔ patient02_seg.nii

3.3 Required Model File

The software requires a clustering model file named kmeans_clustering_model.pkl placed in the same directory as the executable.

4. Software Operation Guide

4.1 Launching the Software

4.1. From Executable

Double-click VPI_Pipeline.exe

4.2 Processing Modes

4.2.1 Single Case Processing

1. Select "**Single Case Processing**" radio button
2. Click "**Browse CT**" to select CT file
3. Click "**Browse Mask**" to select corresponding mask file
4. Configure output directory (default: ~/VPI_Pipeline_Output)
5. Click "**START PROCESSING**"

4.2.2 Batch Processing

1. Select "**Batch Processing**" radio button
2. Click "**Browse Directory**" to select input directory
3. Click "**Scan Directory for Files**" to identify matching pairs
4. Review detected file pairs in the list
5. Configure output directory
6. Click "**START PROCESSING**"

4.3 Common Settings

- **Output Directory**: Location where results will be saved
- **Clustering Model**: Path to the K-means clustering model (default: kmeans_clustering_model.pkl)

5. Processing Pipeline

5.1 Step 1: Image Preprocessing

- **Resampling**: Images resampled to isotropic $1mm^3$ voxel spacing
- **Normalization**: CT values normalized using lung window settings (width: 1500 HU, level: −500 HU)
- **Alignment**: Mask registration to CT image space
- **Output**: Resampled and normalized images saved in 1_resampled/ directory

5.2 Step 2: SLIC Feature Extraction

- **Algorithm**: 3D Simple Linear Iterative Clustering (SLIC)
- **Features Extracted**:
    - Mean, Median, Standard Deviation
    - Variance, Skewness, Kurtosis
    - Quartiles (Q1, Q3), Interquartile Range (IQR)
    - Energy
- **Output**: SLIC features saved in 2_slic_features/ directory

5.3 Step 3: Texture Feature Clustering

- **Method**: K-means clustering with 3 clusters
- **Input**: 10 statistical features from SLIC analysis
- **Output**:
    - Three tissue texture clusters with individual masks
    - Clustering results saved in 3_clustering_results/ directory

5.4 Step 4: Radiomics Feature Extraction

- **Framework**: PyRadiomics implementation
- **Feature Classes**:
    - First-order statistics

- Gray Level Co-occurrence Matrix (GLCM)
- Gray Level Dependence Matrix (GLDM)
- Gray Level Run Length Matrix (GLRLM)
- Gray Level Size Zone Matrix (GLSZM)
- Neighboring Gray Tone Difference Matrix (NGTDM)

- **Settings**: Bin size: 25, BSpline interpolation
- **Output**: 106 radiomics features per cluster saved in 4_radiomics_features/ directory

5.5 Step 5: VPI Prediction

- **Model**: Logistic regression with standardized features
- **Key Prediction Features**:
  1. Habitat1_Firstorder_Skewness
  2. Habitat1_GLDM_SmallDependenceEmphasis
  3. Habitat2_NGTDM_Busyness
  4. Habitat3_GLCM_HabitatProminence
  5. Habitat3_NGTDM_Contrast
- **Prediction Formula**:
  Linear predictor = -1.032 - 0.706×Z1 + 0.504×Z2 - 1.234×Z3 - 0.712×Z4 - 0.826×Z5;
  Probability = 1 / (1 + exp(-Linear predictor))
- **Decision Rule**:
  - Probability > 0.476 = VPI Positive (Class 1)
  - Probability ≤ 0.476 = VPI Negative (Class 0)
- **Output**: Prediction results saved in 5_prediction_results/ directory

---

6. Output Structure

text

```
VPI_Pipeline_Output/
└── CaseName/
    ├── 1_resampled/
    │   ├── CaseName_resampled.nii.gz
    │   ├── CaseName_resampled_mask.nii.gz
    │   └── CaseName_resample_norm.nii.gz
    ├── 2_slic_features/
    │   ├── CaseName_slic_data.pkl
    │   └── CaseName_slic_features.csv
    ├── 3_clustering_results/
    │   ├── CaseName_cluster1.nii.gz
    │   ├── CaseName_cluster2.nii.gz
    │   ├── CaseName_cluster3.nii.gz
    │   └── CaseName_clustering_results.csv
    ├── 4_radiomics_features/
    │   ├── radiomics_params.yaml
    │   └── CaseName_radiomics_features.xlsx
    └── 5_prediction_results/
        ├── CaseName_prediction_results.csv
```

└── CaseName_prediction_results.xlsx

6.1 Output File Descriptions

6.1.1 Processed Images (1_resampled/)

- *_resampled.nii.gz: Resampled CT image (1mm isotropic spacing)
- *_resampled_mask.nii.gz: Resampled mask aligned with CT
- *_resample_norm.nii.gz: Normalized CT image (0-1 range)

6.1.2 SLIC Features (2_slic_features/)

- *_slic_data.pkl: SLIC segmentation labels and image metadata (Python pickle format)
- *_slic_features.csv: Statistical features for each supervoxel (CSV format)

6.1.3 Clustering Results (3_clustering_results/)

- *_cluster[0-2].nii.gz: Binary masks for each texture cluster (NIfTI format)
- *_clustering_results.csv: Cluster assignments for each supervoxel (CSV format)

6.1.4 Radiomics Features (4_radiomics_features/)

- radiomics_params.yaml: PyRadiomics parameter configuration (YAML format)
- *_radiomics_features.xlsx: Complete radiomics features for each cluster (Excel format)

6.1.5 Prediction Results (5_prediction_results/)

- *_prediction_results.csv: Tabular prediction results (CSV format)
- *_prediction_results.xlsx: Excel format with additional formatting

---

7. Results Interpretation

7.1 Prediction Results Table

Column	Description	Format
Case	Patient identifier	String
Probability	VPI probability	0.000-1.000
Prediction	Binary prediction	"VPI Positive" or "VPI Negative"
Cutoff	Decision threshold	0.476
Linear Score	Raw linear predictor	Decimal value
Timestamp	Processing date and time	YYYY-MM-DD HH:MM:SS

7.2 Feature Values Table

Column	Description	Format
Feature	Feature name	String
Cluster	Cluster assignment	"Cluster 0", "Cluster 1", or "Cluster 2"
Original Value	Raw feature value	Decimal or "N/A"
Normalized Value	Z-score normalized value	Decimal

7.3 Interpretation Guidelines

- **VPI Positive**: Probability > 0.476 - High likelihood of visceral pleural invasion
- **VPI Negative**: Probability ≤ 0.476 - Low likelihood of visceral pleural invasion
- **Probability Range**: Higher values indicate greater confidence in VPI prediction
- **Feature Values**: Normalized values indicate deviation from training dataset mean

8. Disclaimer

8.1 Intended Use

This software **at the current stage** is primarily intended for research purposes. While it can provide valuable clinical references, it should be integrated with comprehensive clinical evaluation rather than used as the sole basis for clinical decision-making.

8.2 Limitations

- Requires accurate CT images and tumor masks
- Performance may vary with different patient populations
- Not validated for all CT scanner models and protocols

8.3 Liability

The developers and institutions are not liable for any clinical decisions made based on software output. Users assume full responsibility for proper use and interpretation of results.

8.4 Data Privacy

- All processing occurs locally on user's computer
- No data transmission to external servers
- Users responsible for compliance with local data privacy regulations

9. Continuous Improvement

We are committed to the ongoing validation and improvement of this tool. Users are encouraged to:

- Provide feedback on clinical utility and performance
- Participate in validation studies
- Report any discrepancies with clinical outcomes
- Share suggestions for enhancement

10. Software Interface

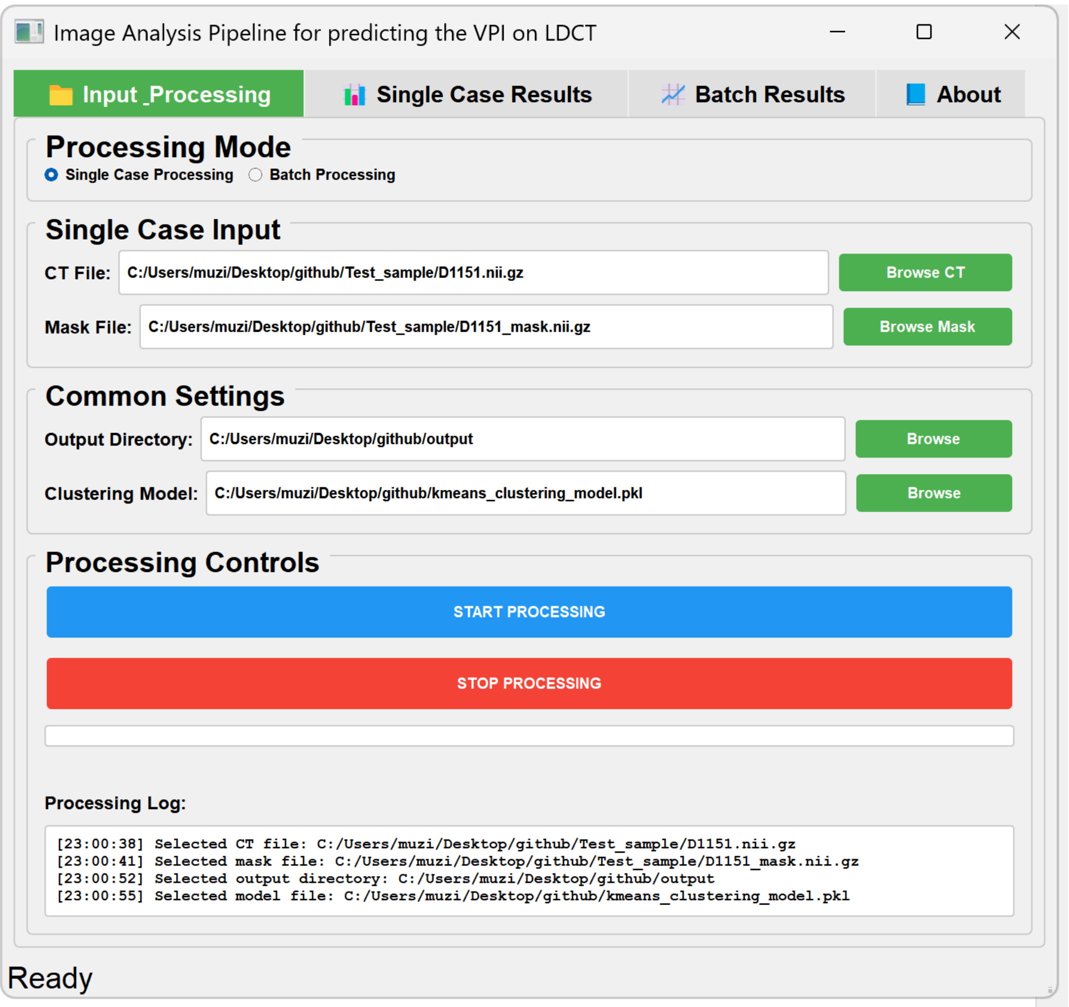

11. Software Output

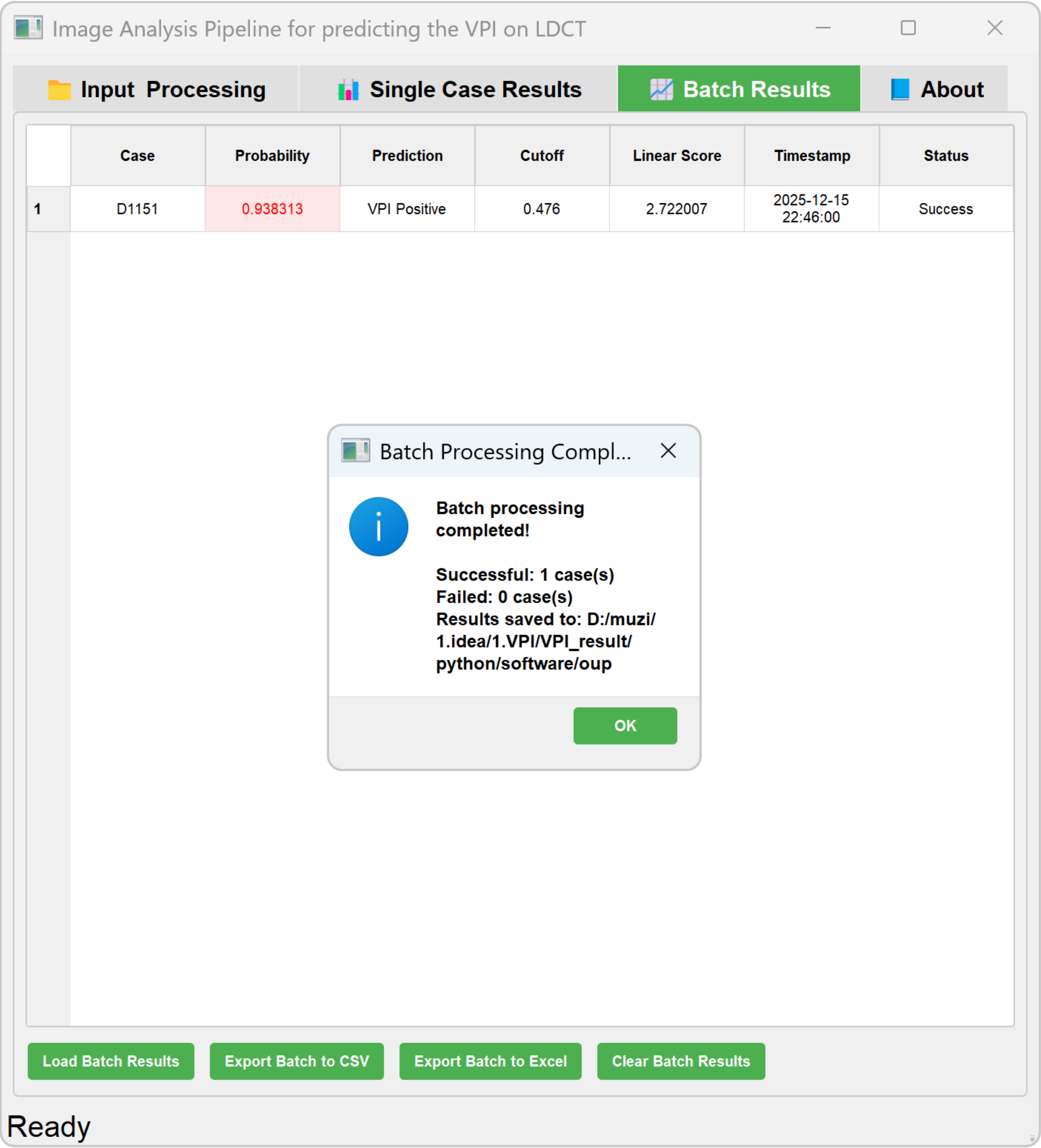
Image Analysis Pipeline for predicting the VPI on LDCT
Input Processing
Single Case Results
Batch Results
About
Case
Probability
Prediction
Cutoff
Linear Score
Timestamp
Status
1
D1151
0.938313
VPI Positive
0.476
2.722007
2025-12-15 22:46:00
Success
Batch Processing Compl...
Batch processing completed!
Successful: 1 case(s)
Failed: 0 case(s)
Results saved to: D:/muzi/1.idea/1.VPI/VPI_result/python/software/oup
OK
Load Batch Results
Export Batch to CSV
Export Batch to Excel
Clear Batch Results
Ready
